# Author Correction: A Tunguska sized airburst destroyed Tall el-Hammam a Middle Bronze Age city in the Jordan Valley near the Dead Sea

**DOI:** 10.1038/s41598-022-06266-9

**Published:** 2022-02-22

**Authors:** Ted E. Bunch, Malcolm A. LeCompte, A. Victor Adedeji, James H. Wittke, T. David Burleigh, Robert E. Hermes, Charles Mooney, Dale Batchelor, Wendy S. Wolbach, Joel Kathan, Gunther Kletetschka, Mark C. L. Patterson, Edward C. Swindel, Timothy Witwer, George A. Howard, Siddhartha Mitra, Christopher R. Moore, Kurt Langworthy, James P. Kennett, Allen West, Phillip J. Silvia

**Affiliations:** 1 Geology Program, School of Earth and Sustainability, Northern Arizona University, Flagstaff, AZ 86011 USA; 2 Center of Excellence in Remote Sensing Education and Research, Elizabeth City State University, Elizabeth City, NC 27909 USA; 3 Department of Natural Sciences, Elizabeth City State University, Elizabeth City, NC 27909 USA; 4 Materials and Metallurgical Engineering, New Mexico Institute On Mining & Technology, Socorro, NM 87801 USA; 5 Los Alamos National Laboratory (Retired), Los Alamos, NM 87545 USA; 6 Analytical Instrumentation Facility, North Carolina State University, Raleigh, NC 27695 USA; 7 EAG Laboratories, Eurofins Materials Science, Raleigh, NC 27606 USA; 8 Department of Chemistry and Biochemistry, DePaul University, Chicago, IL 60614 USA; 9 Geophysical Institute, University of Alaska Fairbanks, 903 Koyukuk Drive, College, AK 99775 USA; 10 Faculty of Science, Charles University, Albertov 6, 12843 Prague, Czech Republic; 11 Southern Research Institute, 757 Tom Martin Drive, Birmingham, AL 35211 USA; 12 US Navy, NAVFAC Mid-Atlantic Region, NS Norfolk, VA 23511 USA; 13 Comet Research Group, Prescott, AZ 86301 USA; 14 Restoration Systems, L.L.C., Raleigh, NC 27604 USA; 15 Department of Geological Sciences, East Carolina University, Greenville, NC 27858 USA; 16 Savannah River Archaeological Research Program, South Carolina Institute of Archaeology and Anthropology, University of South Carolina, New Ellenton, SC 29809 USA; 17 CAMCOR, University of Oregon, 1443 E 13th Ave, Eugene, OR 97403 USA; 18 Department of Earth Science and Marine Science Institute, University of California, Santa Barbara, CA 93106 USA; 19 College of Archaeology, Trinity Southwest University, Albuquerque, NM 87109 USA

Correction to: *Scientific Reports* 10.1038/s41598-021-97778-3, published online 20 September 2021

The original version of this Article contained errors.

Some of the figure panels have been manipulated to remove the features irrelevant to the scientific content depicted in those (e.g. measuring tape, previous image labels, visible fingers etc.). The Authors recognise that this level of manipulation was inappropriate, and provide original images. As a result, the following panels were corrected and the original versions of the figures containing these panels are shown below for the record: Figure 3, Figures 4C & 4D, Figures 7C & 7D, Figures 8A & 8C, Figures 10A-C, Figures 11A-C, Figures 13D, 13F & 13H, Figure 14A & 14C, Figures 15A & 15B, Figures 18A & 18D, Figure 21A, Figures 22A, 22C & 22E, Figures 23A, 23C & 23F, all panels in Figure 25, Figures 26A & 26B, all panels in Figure 28, Figures 29B, 29D, and 29E, all panels in Figure 30, Figure 31A, Figure 32A, 32C, 32D & 32F, Figures 33A & 33B, all panels in Figure 34, Figures 38B-D, Figure 39A, Figure 40A, Figures 41A & 41C, Figures 42A & 42C, Figure 43, Figure 44C, Figure 45C, Figure 46C, and Figure 47B & 47C.

Additionally, scales were re-adjusted in the following panels: Figure 4C, Figures 11A, 11C & 11D, and Figure 26A. A number of panels was rotated 180 degrees so that original labels are not oriented upside down; this affects Figure 13H, Figure 22B, Figures 25C, 25E, 25F, & 25K, Figure 32A & 32B, Figure 34E, Figures 38C & 38F. Panels 14A & 14B had colour adjusted – these panels now show original images. Position of panels in Figure 51 was re-adjusted.

Finally, panel 15b was horizontally flipped in relation to the original and had the arrow pointing north obscured. It has now been replaced with a correct image.

Original versions of all affected figures are reproduced below for the record.

**Figure 3:**
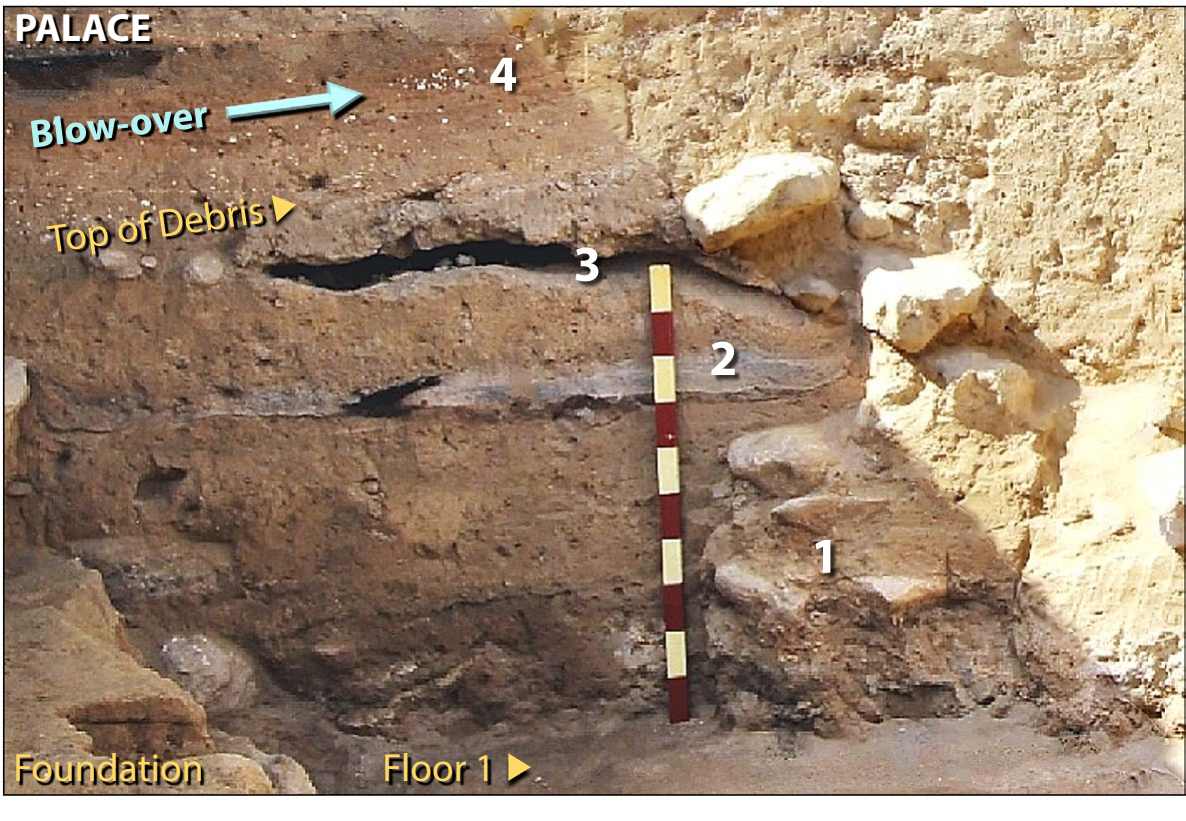
Destruction of the multi-storied palace. Photo showing jumbled rubble of four-story Palace atop Floor 1. Remains of debris from upper stories are labeled as follows. #1 represents broken mudbricks and debris from shattered upper walls. #2 and #3 are voids and layers formed when trapped textiles (rugs and tapestries) burned, leaving only fibrous ash and carbon. ‘Blow-over’ (in blue) is composed of windblown, laminated deposits that sealed the ruined structure for ~ 3600 years, beginning at the time of destruction. #4 marks fragments of white limestone plaster (CaCO_3_) mixed with carbonate spherules from the palace walls and ceiling. Scale stick has 10-cm markings.

**Figure 4:**
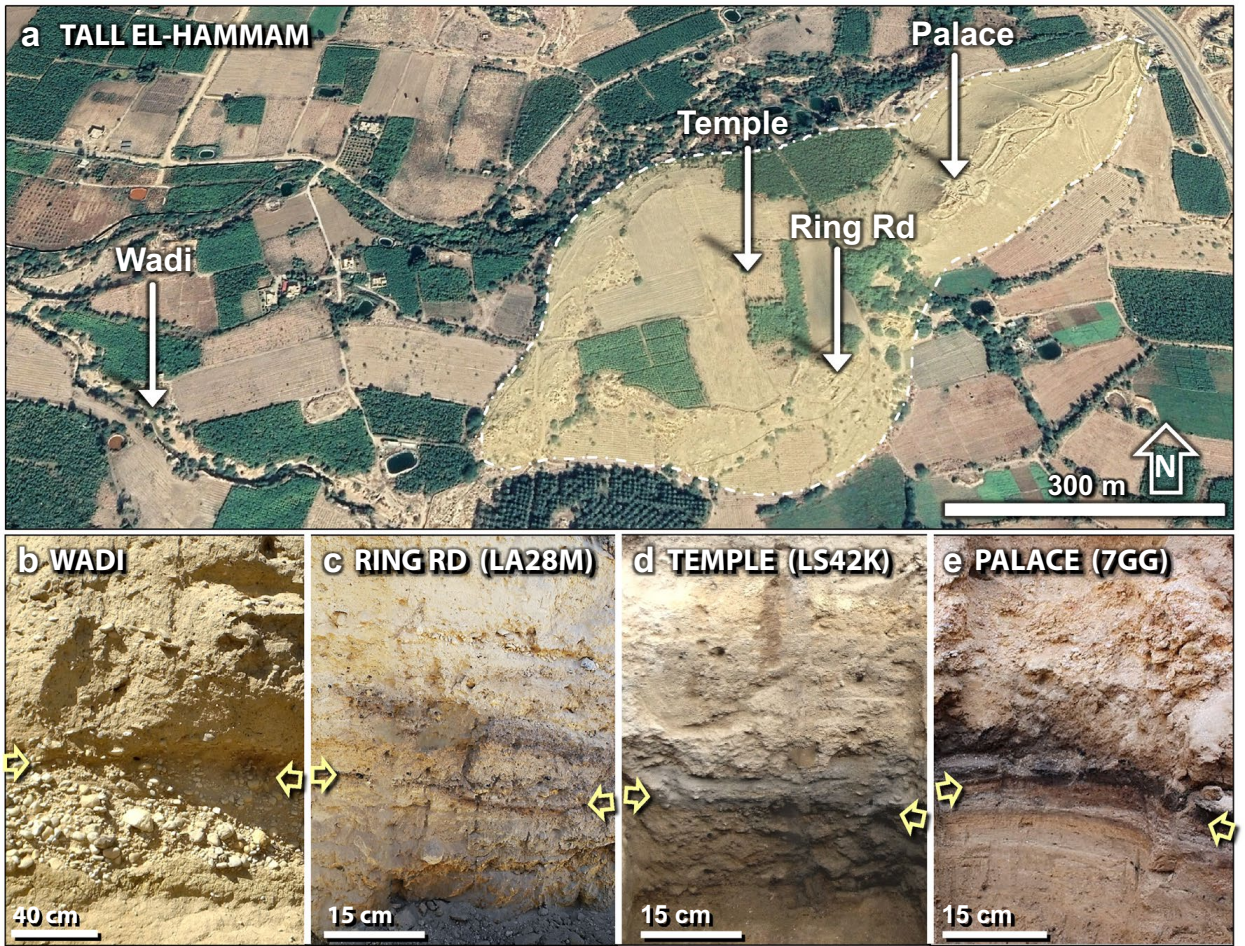
Sedimentary profiles. (**a**) Map of the city (white dashing line) showing sampling locations, spanning ~ 1100 m. Source of base image: “Tall el-Hammam”. 31° 50.483 N, 35° 40.029 E. Google Earth; CNES/Airbus. Imagery date: 11/26/2019; accessed: 4/4/2021. (**b**) The wadi; (**c**) the ring road in Field LA; (**d**) the palace in Field LS; and (**e**) the palace in Field UA. In the wadi, yellow arrows mark the level of the 3600-year-old stratum. Inside the city, the arrows mark the location of the charcoal-and-ash-rich dark layer.

**Figure 7:**
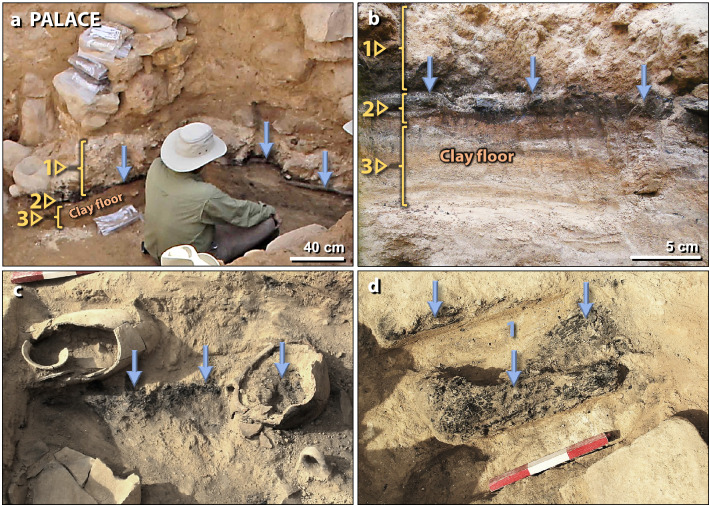
Destruction layer in the palace. (**a**) Photo of excavation in an exterior food preparation area of the palace. #1 marks MB II debris that was most likely deposited by post-fire erosion. #2 points to charcoal-rich ‘dark layer’ indicating a major fire in the palace. Contains fragments of plaster and limestone spherules. Blue arrows mark its top. #3 points to the cross-section of excavated clay flooring. (**b**) Close-up photo of the same palace sampling sequence as in panel ‘**a**’. (**c**) Photo of broken pots with carbonized grains embedded in MB II 1.5-m-thick debris matrix, mostly composed of pulverized mudbrick and plaster fragments and limestone spherules. Debris matrix is found in the space between all palace walls. Note charcoal inside the broken pot. The end of a scale stick with 10-cm divisions is at upper left. (**d**) Charred palace roof timber surrounded by 1.5-m-thick charcoal-rich debris matrix of pulverized mudbrick. A scale stick shows 10-cm markings.

**Figure 8:**
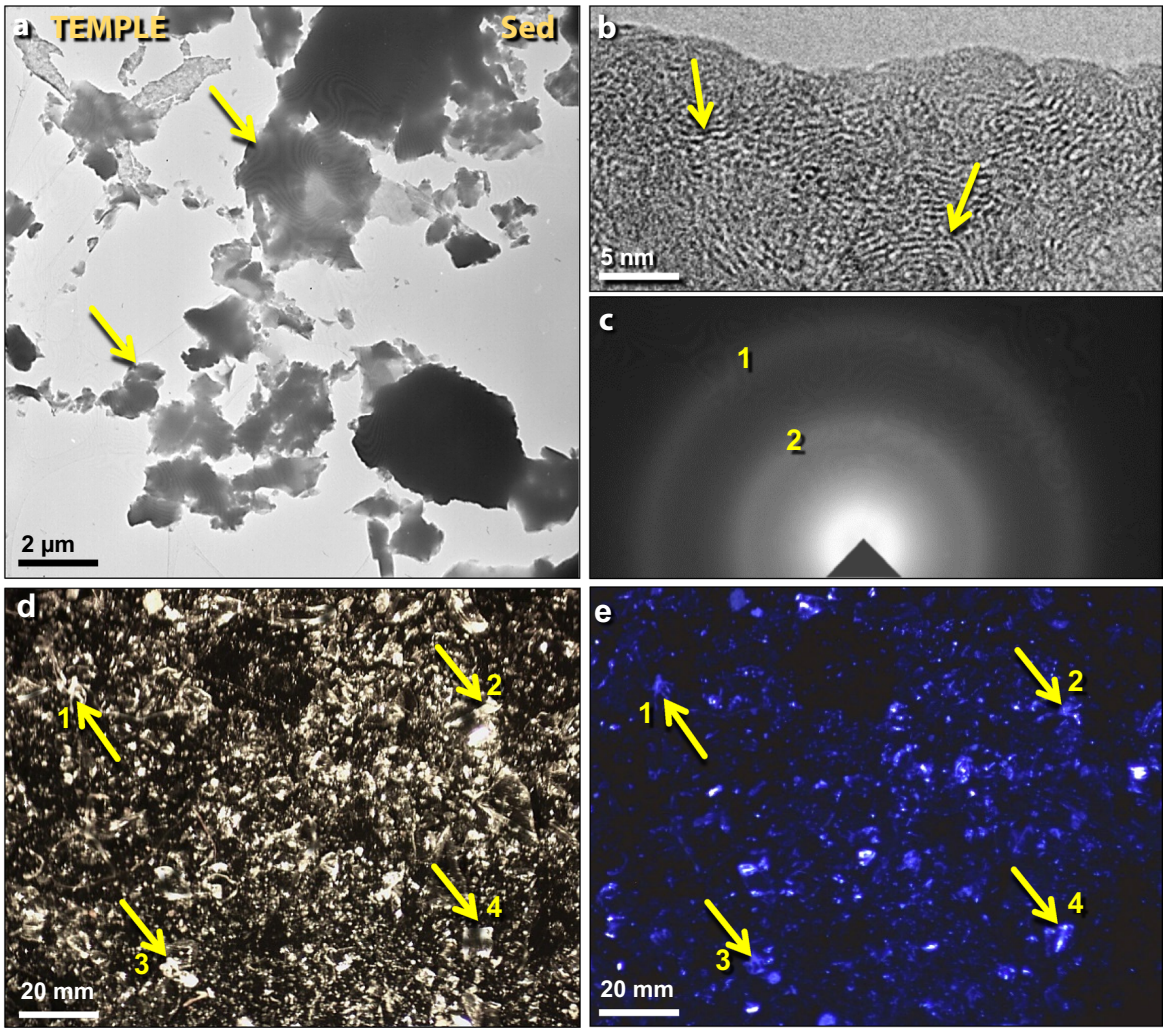
Diamonoids (diamond-like carbon) in temple sediment. (**a**) Transmission electron microscopy (TEM) image of clusters of amorphous diamonoids. (**b**) Bright-field high-resolution transmission electron microscopy (HRTEM) of acid-resistant residue showing the short-range ordering of carbon atoms. (**c**) Selected-area electron diffraction (SAD) of residue and grid film, confirming that the residue is amorphous carbon. (**d**) Photomicrograph of diamonoids showing white to clear material on black carbon SEM tab; (**e**) photomicrograph of the same area as in panel ‘**d**’ showing that the diamonoids luminesce at ~ 440 nm, typical of cubic diamonds.

**Figure 10:**
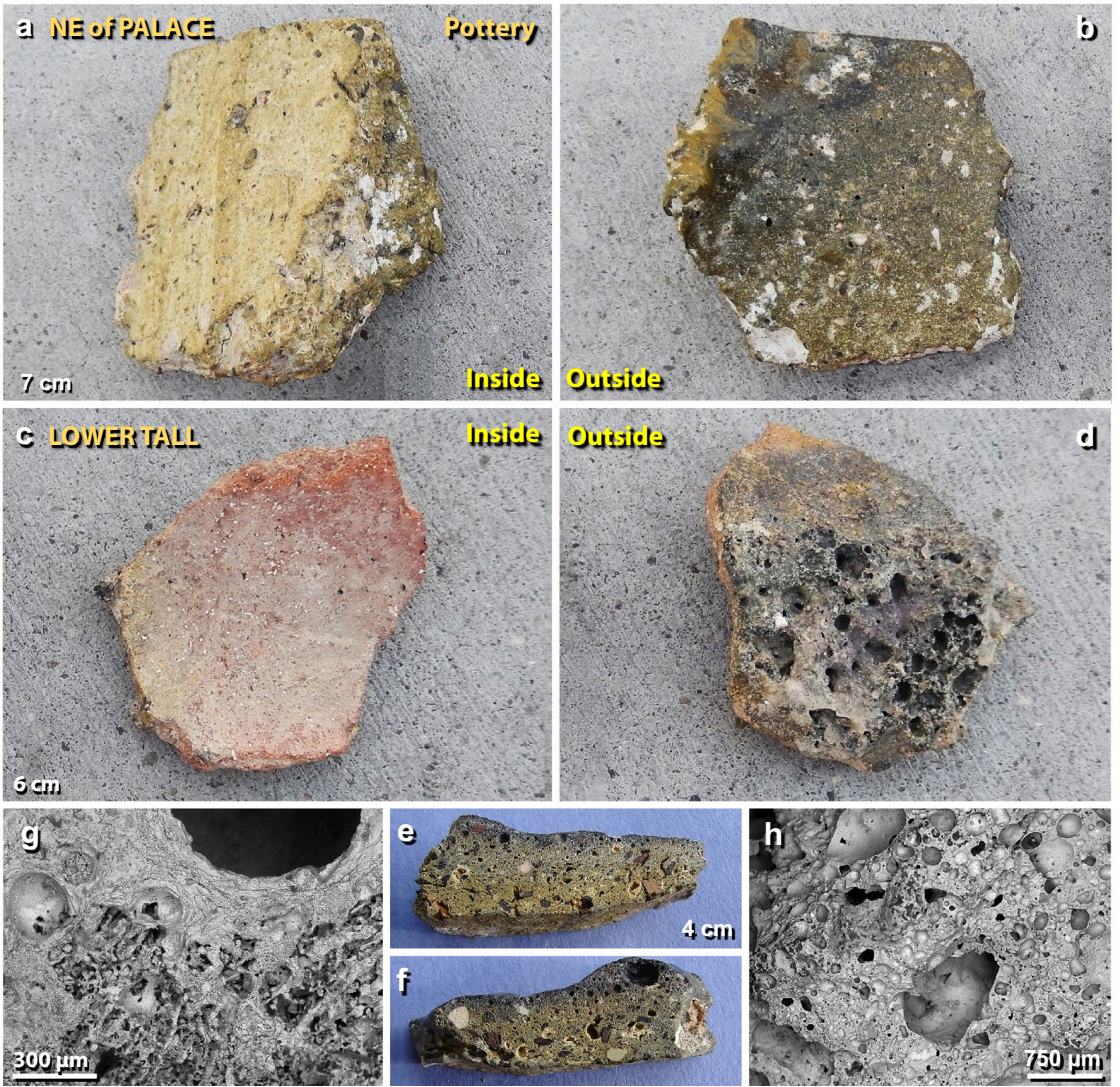
Melted pottery. (**a**) Photos of a 7-cm-wide potsherd from a broken storage jar from NE of the palace, showing unmelted inner surface and (**b**) the darker melted outer surface of the potsherd. The upper-left edge in panel ‘**b**’ is the outward-curved lip of a storage jar. (**c**) Potsherd of a 6-cm-wide storage jar from the lower tall, displaying an unaltered inner surface, and (**d**) the highly vesicular outer surface. (**e**)–(**f**) Photos of both edges of the sliced section of sherd in panels ‘**a**’ and ‘**b**’ above. (**g**)–(**h**) SEM images of the highly vesicular sliced surface of sherd in panels ‘**a**’ and ‘**b**’.

**Figure 11:**
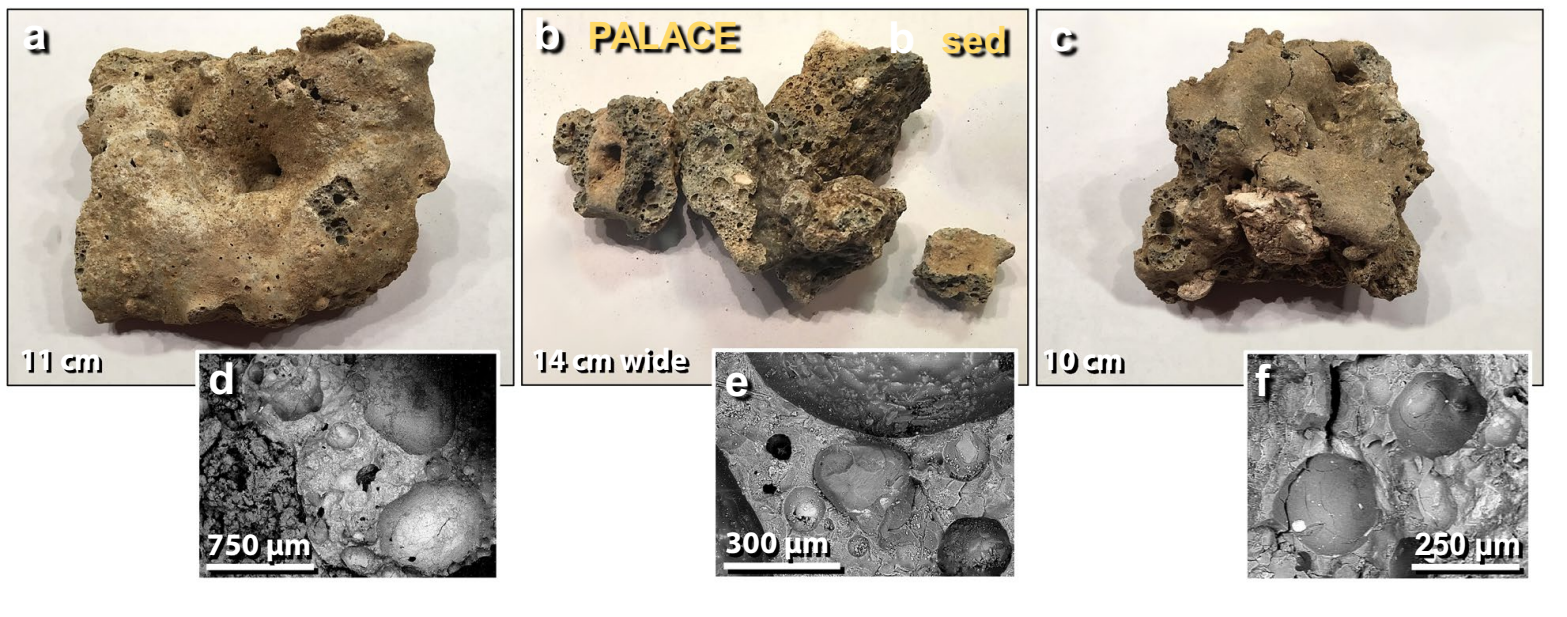
Melted mudbrick from the palace. (**a**) The upper surface of meltglass, showing non-vesicular ‘skin’; (**b**) broken surfaces of meltglass displaying vesicular texture; (**c**) upper surface and broken faces of meltglass. Note large unmelted light-colored mineral inclusion. (**d**)–(**f**) SEM images of highly vesicular surfaces of broken meltglass. Note bright metallic inclusions in several vesicles.

**Figure 13:**
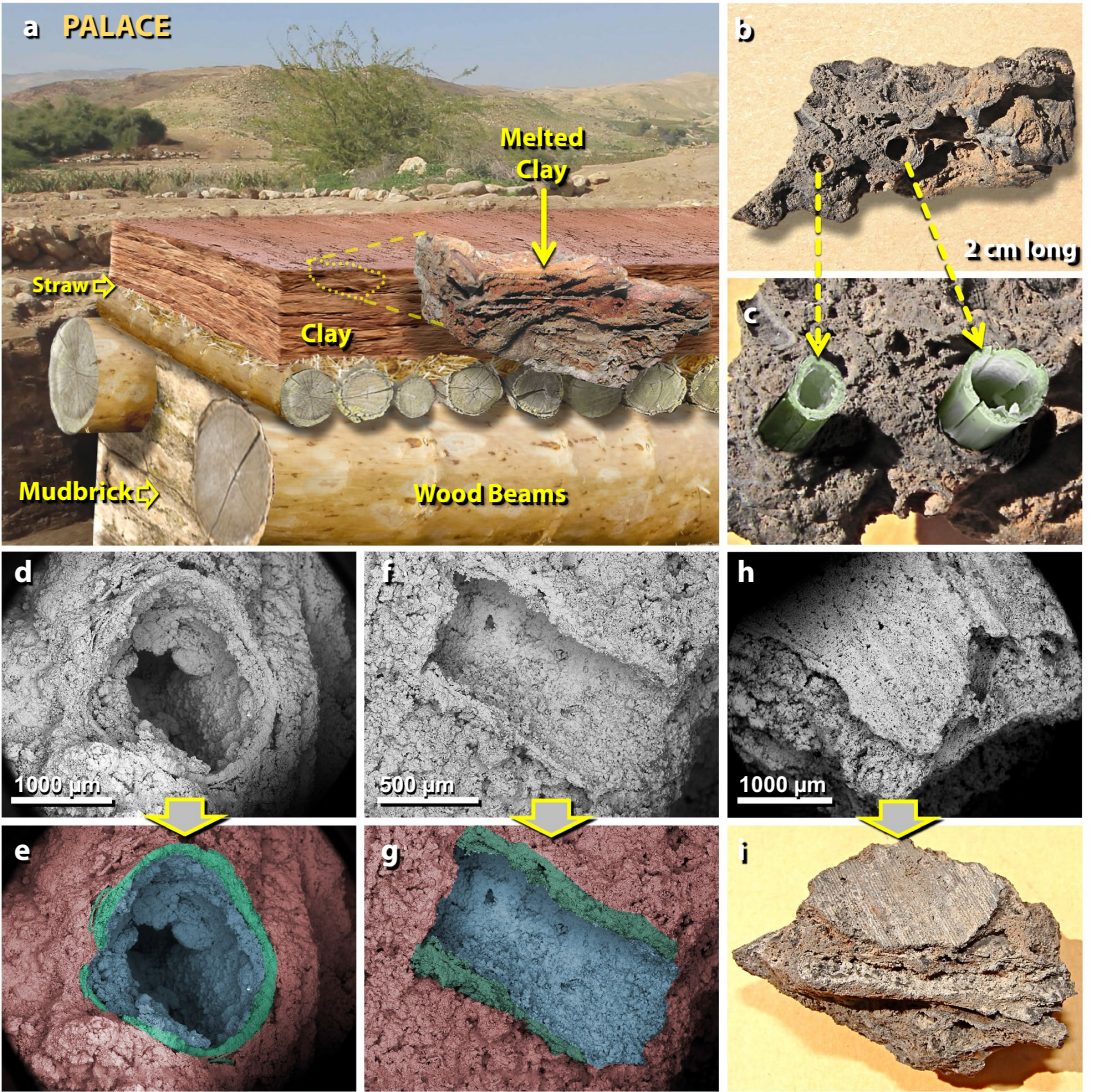
Melted palace roofing clay. (**a**) Artist’s cutaway depiction of typical roof construction at TeH. The construction involved sequentially plastering multiple layers of clay (~ 10 cm or more in total thickness) over a bed of leaves and straw placed over wood beams. “Melted clay” inset at middle right is a photo of melted roofing clay still displaying horizontal layers of clay plaster. (**b**) Fragment of melted roofing clay exhibiting ~ 2-mm-diameter tubular holes left after incineration of straw; (**c**) artist’s depiction, re-creating protruding straw before burning; (**d**) SEM image is the end-view of the hole left by burned straw embedded in roofing clay; (**e**) manually constructed EDS-based phase map of the same image, showing composition as determined by SEM–EDS; red represents melted clay matrix, green represents high-silica glass (60–90 wt.% SiO_2_) formed from melted silicified straw, and blue represents the silica-rich interior of the hole. (**f**) SEM image is the side-view of the imprint left by burned straw; (**g**) manually constructed EDS-based phase map of the same image, color-coded as in the previous example. (**h**) SEM image of leaf imprint into the clay roofing material, showing the ribbed structure of a leaf; (**i**) photomicrograph of the same object.

**Figure 14:**
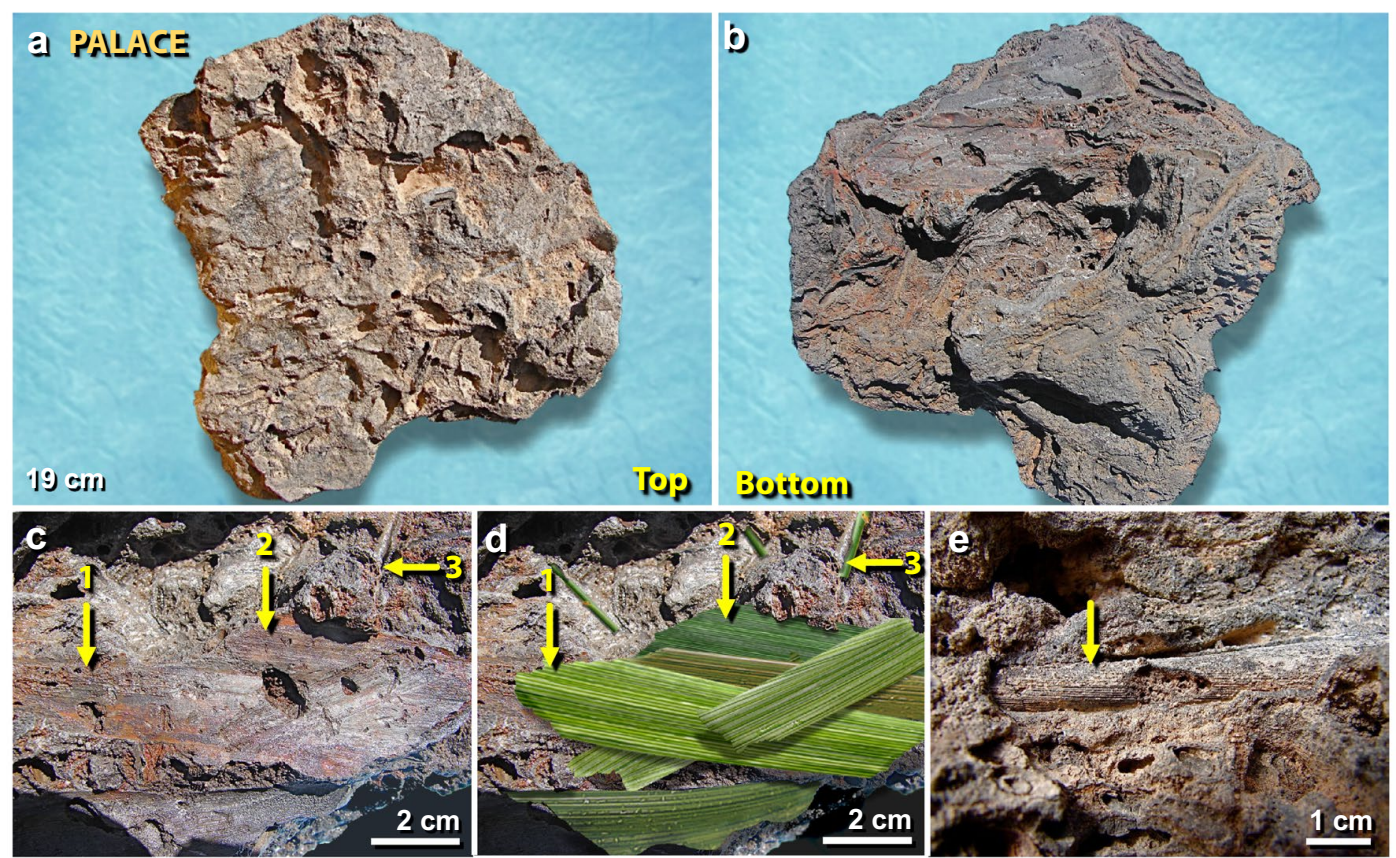
Plant imprints in melted roofing clay from the palace. (**a**) The upper surface of a 19-cm-wide piece of roofing clay, melted and distorted at high temperatures; (**b**) lower surface of the same object, showing imprints of silicified plant material; (**c**) closeup of the lower surface with numbered yellow arrows pointing to ribbed imprints of leaves pressed into the bottom of roofing clay (#1 through #3); (**d**) artist’s depiction, re-creating possible leaf structures before combustion; (**e**) yellow arrow points not to a plant imprint, but rather to a cylinder-like silica-rich pyromorph of plant stem embedded in roofing clay. From the destruction layer in the palace (Field UA, Square 7GG).

**Figure 15:**
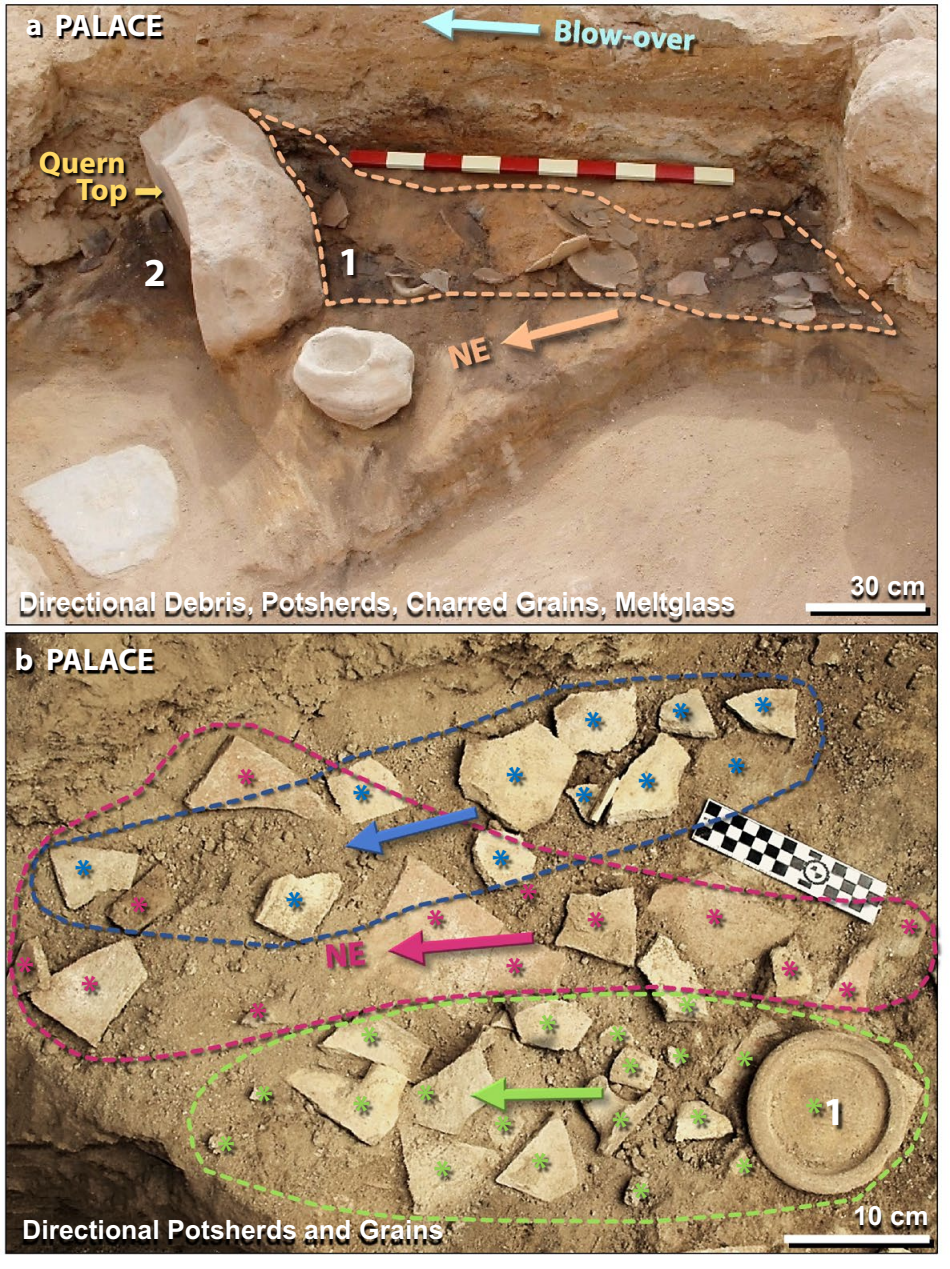
The directionality of mudbricks, potsherds, and grains. (**a**) 400-kg quern, at left, used for grinding grain, is tipped over with top towards NE. Image view spans ~ 2.5 m. Area #1 shows broken pottery and meltglass piled against quern from SW to NE (arrow). Area #2 contains charcoal, charred grains, ash, and mudbrick fragments, but no potsherds, suggesting that the quern shielded the floor to the NE. The area at the top labeled ‘blow-over’ is evidence that strong winds sealed the deposit with windblown laminated material that includes pulverized mudbrick, charcoal, ash, and fragments of white plaster. The draping of the blow-over indicates debris traveled from SW to NE. The scale stick is in 10-cm intervals. (**b**) Directional potsherds. Blue, red, and green asterisks (*) represent color-coded potsherds from three different pots. Arrows mark the motion of potsherds from SW to NE, spanning ~ 1 m. Area #1 represents the inverted bottom of the pot with its smaller fragments strewn to the left. Some pots contained charred grains also strewn in a SW-to-NE direction. Radiocarbon dates on charred grains confirm an age of ~ 1650 BCE (3600 cal BP).

**Figure 18:**
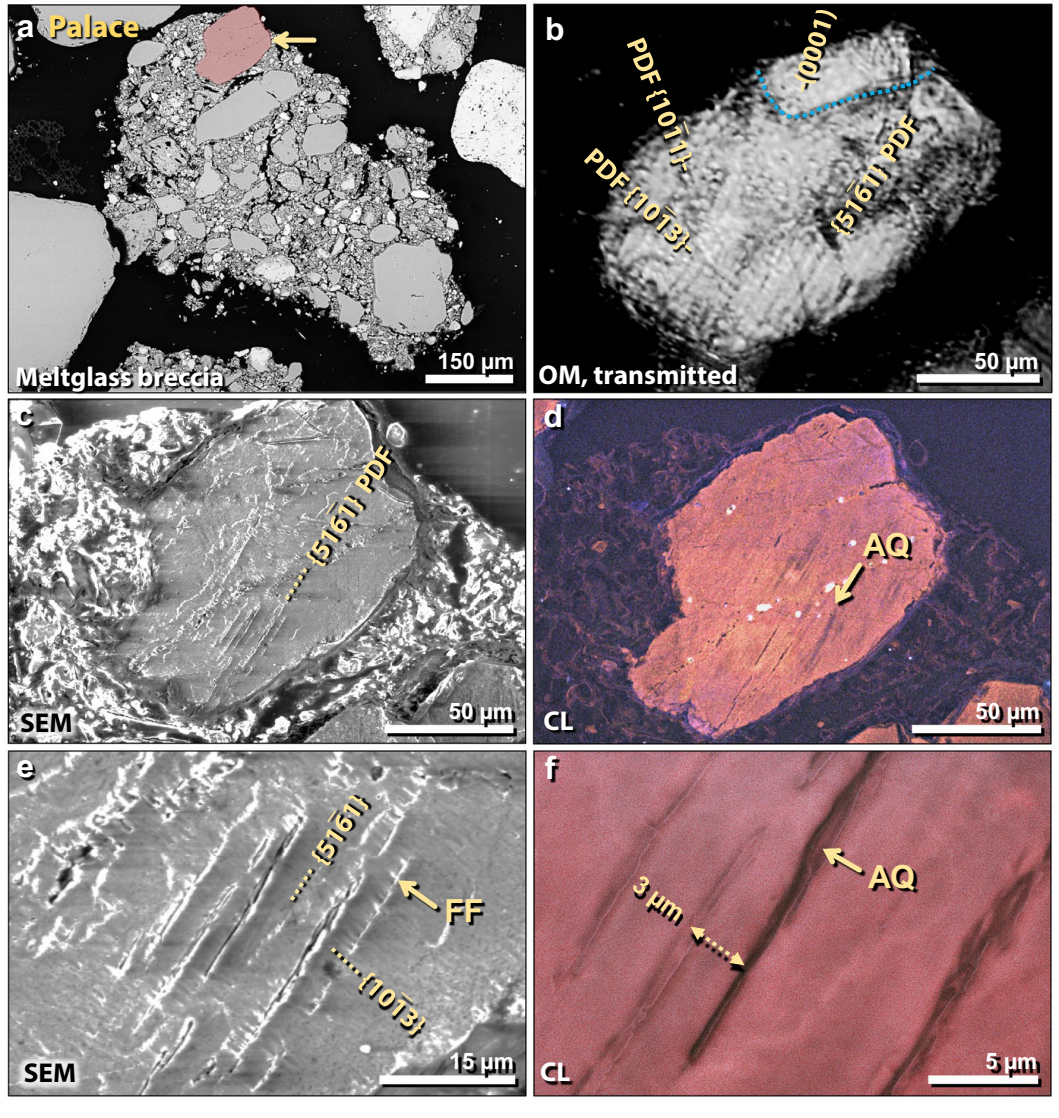
Shocked quartz grain from TeH palace. (**a**) SEM image of a 750-µm-wide fragment of meltglass breccia from the palace, containing melted and partially melted grains surrounded by Ca–Al–Si meltglass. Shocked quartz grain in red at arrow; other grains are not shocked. (**b**) Transmitted light photomicrograph using an optical microscope (OM); 155-µm-wide HF-etched quartz grain is same as in panel ‘**a**’. Lamellae are labeled as Miller-Bravais indices (hkil) (ANIE program^55^). Three sets in lower crystallite are all closely-spaced PDFs crossing the entire crystallite. Blue dotted line marks the boundary of one crystallite in polycrystalline grain, containing only one set of lamellae, the (0001) plane with its pole parallel to the *c* axis. (**c**) SEM image of same HF-etched grain displaying visible lamellae, which excludes them as tectonic lamellae. (**d**) SEM-CL image of same grain showing multiple closely-spaced non-luminescent lamellae (black), indicative of amorphous quartz (AQ). (**e**) Close-up SEM image of same grain, showing visible lamellae, all of which display short-range feather features (FF) that form at ≥ 7 GPa. (**f**) SEM-CL close-up image, showing black non-luminescent centers of lamellae, indicative of amorphous quartz. Lamellar spacing is ~ 3–5 µm.

**Figure 21:**
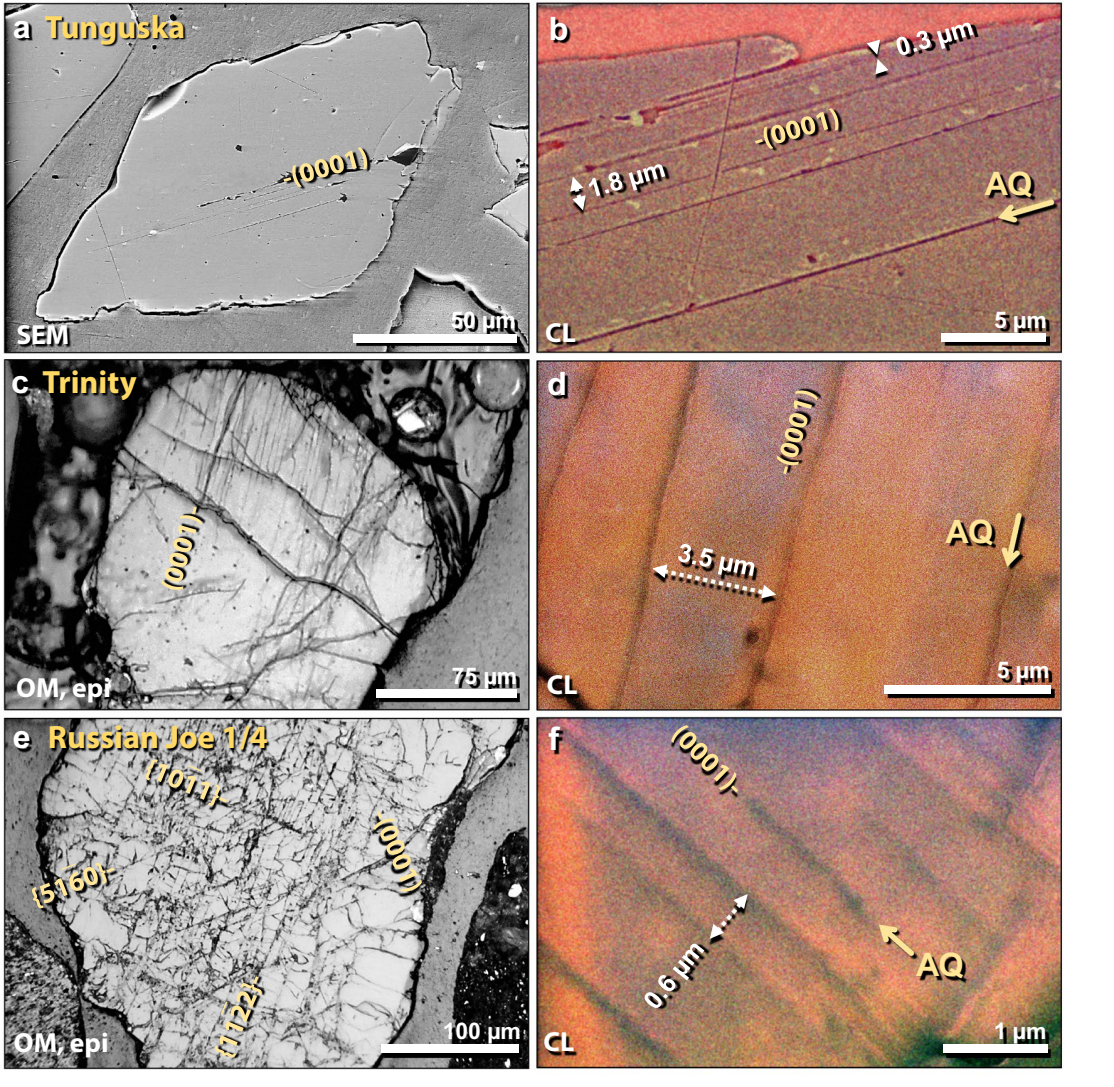
Shocked quartz from known airbursts. (**a**) SEM image of 140-µm-wide shocked quartz grain from Tunguska airburst. Discontinuous lamellae formed along the (0001) plane. (**b**) Close-up SEM-CL image of same grain as in panel ‘**a**’. Lamellae spacing ranges from ~ 0.3 to 5 µm. Black lamellae indicate amorphous quartz (AQ). (**c**) Epi-illumination photomicrograph of 240-µm-wide shocked quartz grain embedded in trinitite that was ejected ~ 400 m from ground zero of the Trinity detonation. Discontinuous, sub-planar, sub-parallel lamellae are oriented along the (0001) plane. (**d**) Close up SEM-CL image of same grain as in panel ‘**c**’. Lamellae spacing ranges from ~ 2.5 to 6.5 µm. Black lamellae indicate amorphous quartz (AQ). (**e**) Epi-illumination photomicrograph of 520-µm-wide shocked quartz grain from Russia's Joe-1/4 nuclear detonations; displays discontinuous sub-planar, sub-parallel micro-fractures and lamellae, just as reported for impact-shocked grains in the Charlevoix crater^56^. There are four sets of lamellae, one of which is oriented parallel to the (0001) plane. (**f**) Close up SEM-CL image of same grain as in panel ‘**e**’. Lamellae spacing ranges from ~ 0.3 to 1.3 µm. Black lamellae indicate amorphous quartz (AQ).

**Figure 22:**
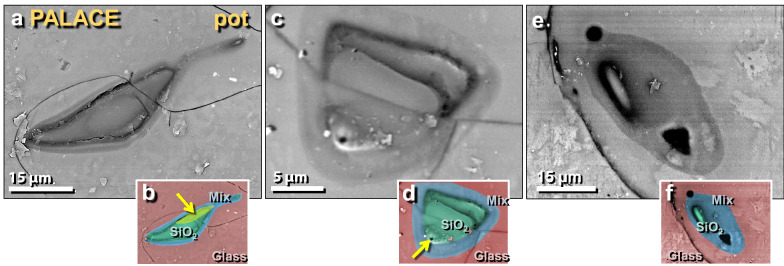
SEM images of melted quartz grains on melted potsherd from the palace. (**a**) Highly melted quartz grain from the upper surface of melted pottery; shows flow lines of molten quartz in darker ‘neck’ at upper right; (**b**) manually constructed EDS-based phase map showing 100% quartz grain (green) embedded in Ca–Al–Si matrix of melted pottery (red); blue marks mixing zone between SiO_2_ and matrix at approximately > 1713 °C, the melting point of quartz. Yellow arrow points to area depleted in oxygen, indicating high-temperature transformation to elemental Si mixed with melted SiO_2_. (**c**) Highly melted quartz grain; (**d**) manually constructed EDS-based phase map showing diffusion/mixing zone in blue with arrow pointing to bubble, indicating outgassing as grain reached temperatures above its melting point. (**e**) Quartz grain that has almost completely melted; (**f**) manually constructed EDS-based phase map showing the small remnant of a melted quartz grain (green) with a wide mixing zone (blue).

**Figure 23:**
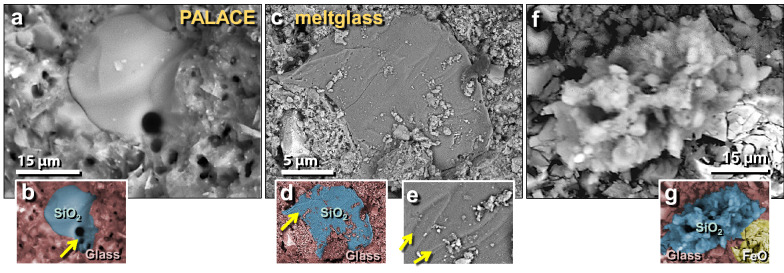
SEM images of melted quartz grains on melted mudbrick from the palace. (**a**) Highly melted quartz grain; (**b**) manually constructed EDS-based phase map indicates center is pure SiO_2_ surrounded by melted mudbrick. Arrow points to vesicles indicating outgassing as grain temperature rose above ~ 1713 °C, the melting point of quartz. (**c**) The surface of a flattened quartz grain showing flow marks toward the upper right. High temperatures are required to lower the viscosity sufficiently for quartz to flow. (**d**) Manually constructed EDS-based phase map with an arrow pointing to vesicles indicating outgassing at high temperatures. (**e**) Close up of grain in panel ‘**c**’ showing flow marks (schlieren) at arrows. (**f**) Shattered, melted quartz splattered onto mudbrick meltglass; (**g**) manually constructed EDS-based phase map indicating that the blue area is SiO_2_; the yellow area is a shattered, thermally altered Fe-oxide grain.

**Figure 25:**
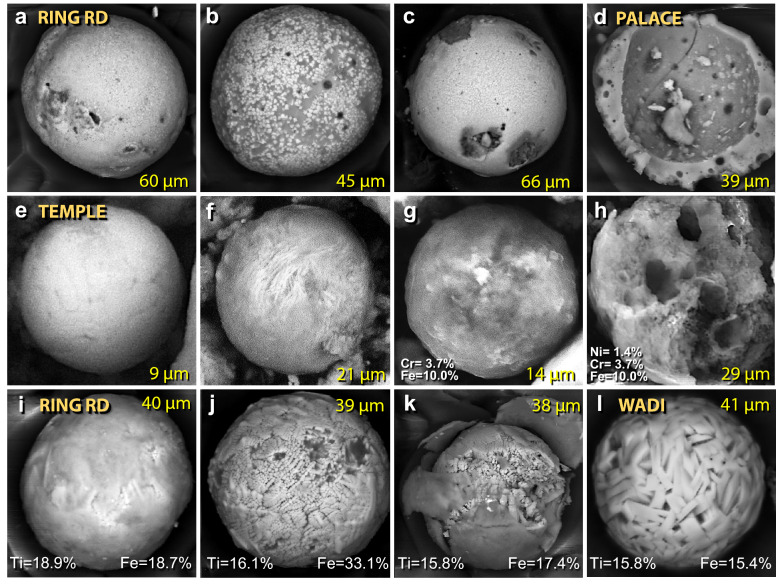
SEM images of mostly silica-rich spherules from TeH. (**a**)–(**d**) Representative spherules from the ring road on the lower tall. SEM images of iron-rich spherules. (**e**)–(**f**) Fe-rich spherules from the temple complex. (**g**) temple spherule containing ~ 3.7 wt.% Cr. (**h**) Broken, vesicular spherule from temple containing 1.4 wt.% Ni and 3.7 wt.% Cr. SEM images of titanium-rich spherules. Ti content of these ranges from 18.9 to 1.2 wt.%, averaging 10.7 wt.%. (**i**)–(**k**) Spherules from the ring road. (**l**) Spherule from the wadi site.

**Figure 26:**
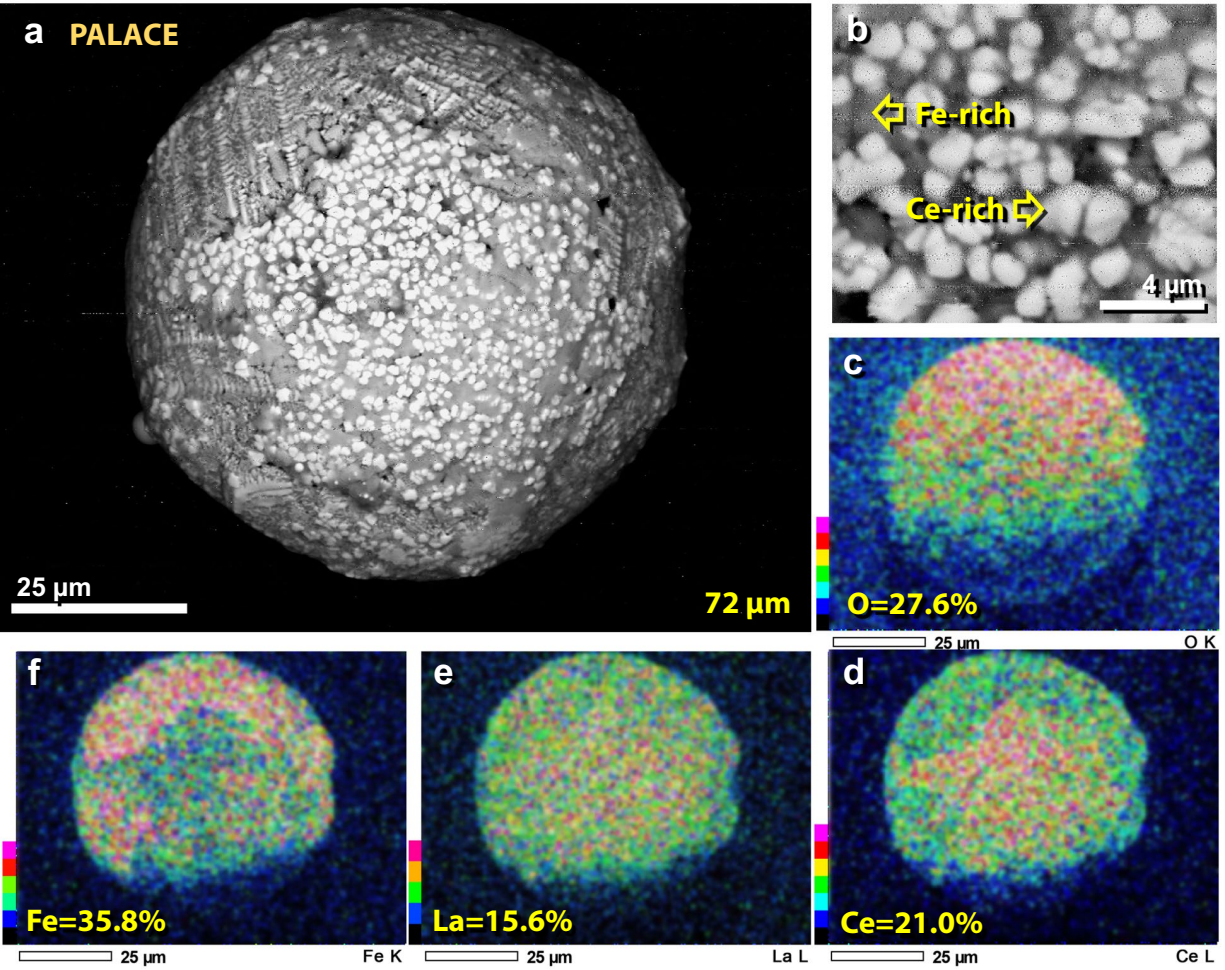
SEM image of rare-earth (REE) spherule. (**a**) REE-rich 72-µm-wide spherule from the palace, dominantly composed of Fe, La, Ce, and O. (**b**) Close up of REE blebs found on the spherule. (**c**)–(**f**) SEM–EDS elemental maps showing composition. La = 15.6 wt.% and Ce = 21.0 wt.%. Ce is enriched over Fe and La in the middle part of the spherule, as seen in panels ‘**d**’ through ‘**f**’.

**Figure 28:**
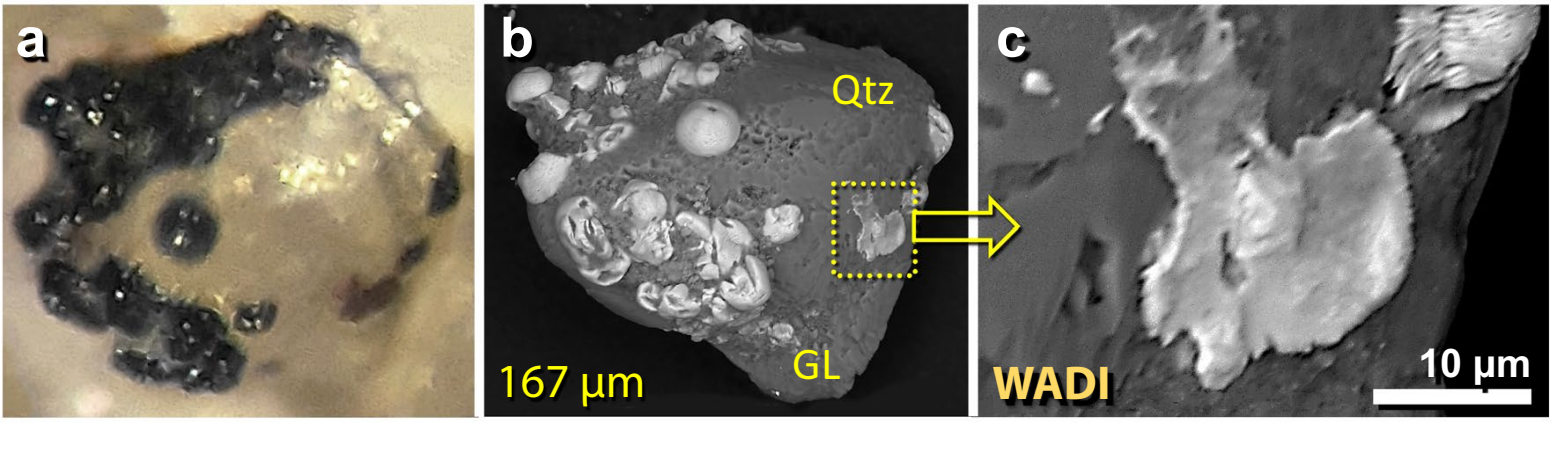
Fe-rich spherules embedded in meltglass. (**a**) Optical photomicrograph of a 167-µm-wide piece of meltglass with embedded Fe-rich spherules. (**b**) SEM image of same grain as in panel ‘**a**’. Melted quartz grain (Qtz) is embedded in Ca–Al–Si-rich matrix, which has the same composition as melted mudbrick. (**c**) SEM close-up image of the boxed area and panel ‘**b**’, showing splattered Fe-rich spherule.

**Figure 29:**
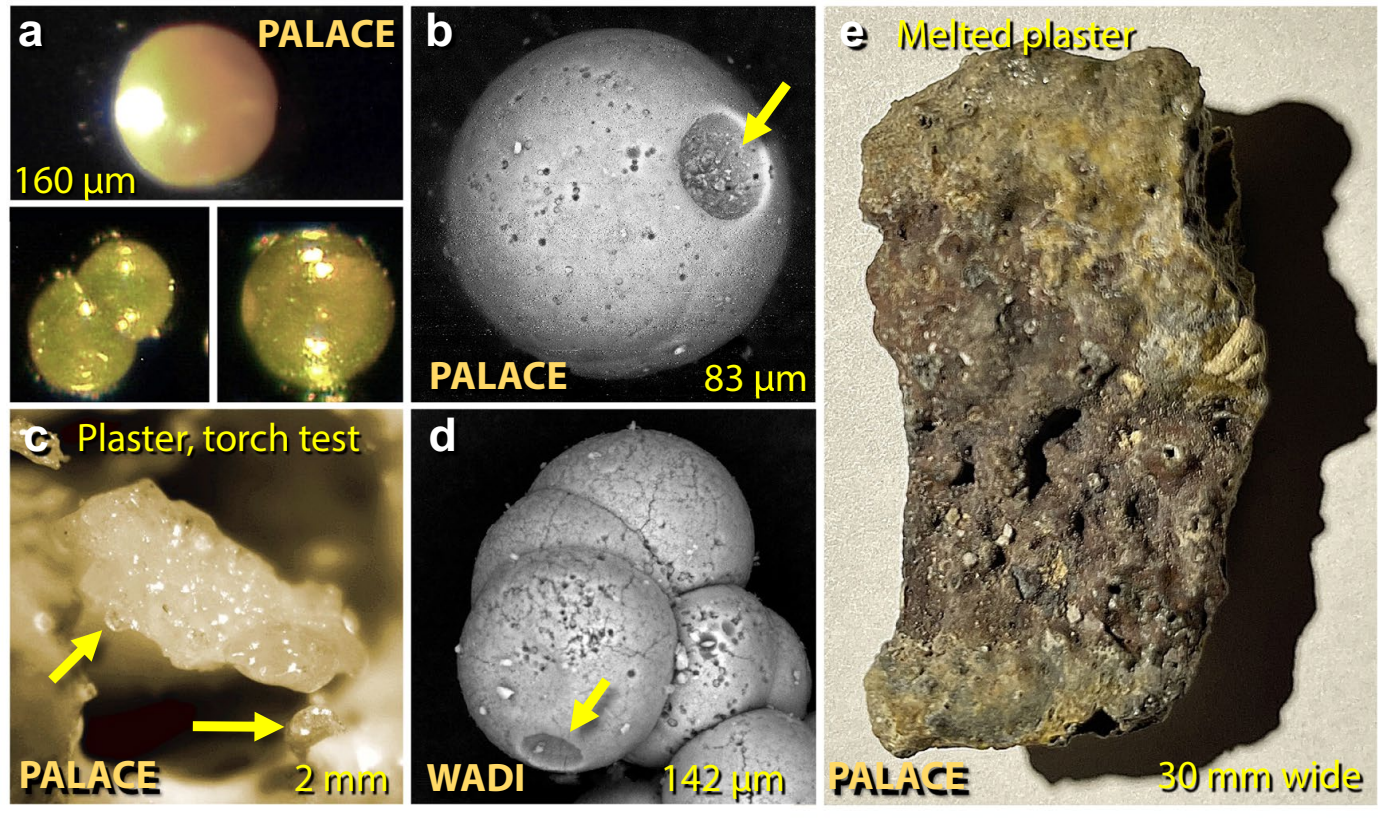
Images of calcium carbonate spherules and melted plaster from TeH. (**a**) Photomicrographs of translucent, amber-colored CaCO_3_ spherules from the destruction layer in the palace. (**b**) SEM image of 83-µm carbonate spherule with impact or outgassing crater at arrow. (**c**) Photomicrograph of ~ 2-mm-wide piece of partially melted palace plaster from oxygen/propylene torch test, showing incipient melting at 1500 °C. Arrows point to hemispheric droplets emerging as spherules. (**d**) 142-µm cluster of 8 carbonate spherules with apparent impact or outgassing crater at arrow. (**e**) 64 × 30 mm piece of melted plaster that broke off the palace wall and became melted. It is composed only of calcium, carbon, and oxygen.

**Figure 30:**
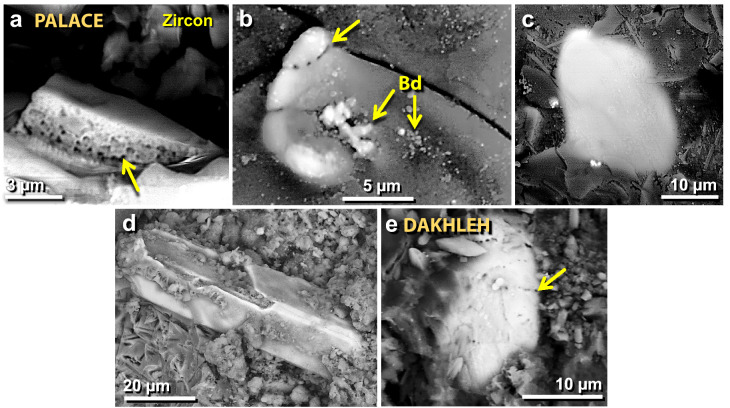
SEM images of melted zircon grains. (**a**) Melted TeH zircon grain with bubbles at yellow arrow due to high-temperature dissociation and/or entrapped porosity. (**b**) Melted TeH zircon grain decorated with bubbles along the fracture line at upper arrow; arrows labeled “Bd” point to bright granular baddeleyite, ZrO_2_, formed during the high-temperature dissociation of zircon. (**c**) Almost fully melted TeH zircon grain mixing into the Ca–Al–Si matrix. (**d**) A typical unmelted zircon grain from TeH with straight, euhedral edges. Grain shows cracks on the top surface from possible thermal or mechanical damage. (**e**) For comparison, from cosmic airburst/impact at Dakhleh Oasis in Egypt: melted zircon decorated with lines of bubbles (arrow).

**Figure 31:**
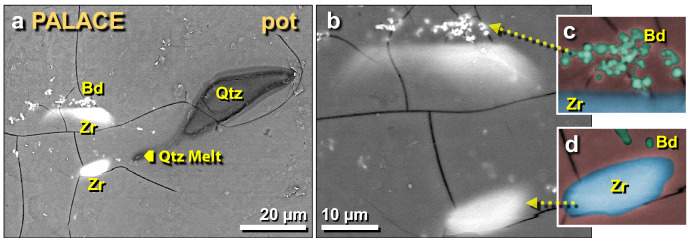
SEM images of other melted zircon grains in palace potsherd. (**a**) Two melted zircon grains adjacent to a previously discussed melted quartz grain; (**b**) close-up of same zircon grains; (**c**) manually constructed EDS-based phase map showing baddeleyite grains in green. The blue area represents melted zircon, while the red background represents the Ca–Al–Si matrix of the melted pottery. (**d**) Manually constructed EDS-based phase map of zircon grain showing small baddeleyite grains in green at the top.

**Figure 32:**
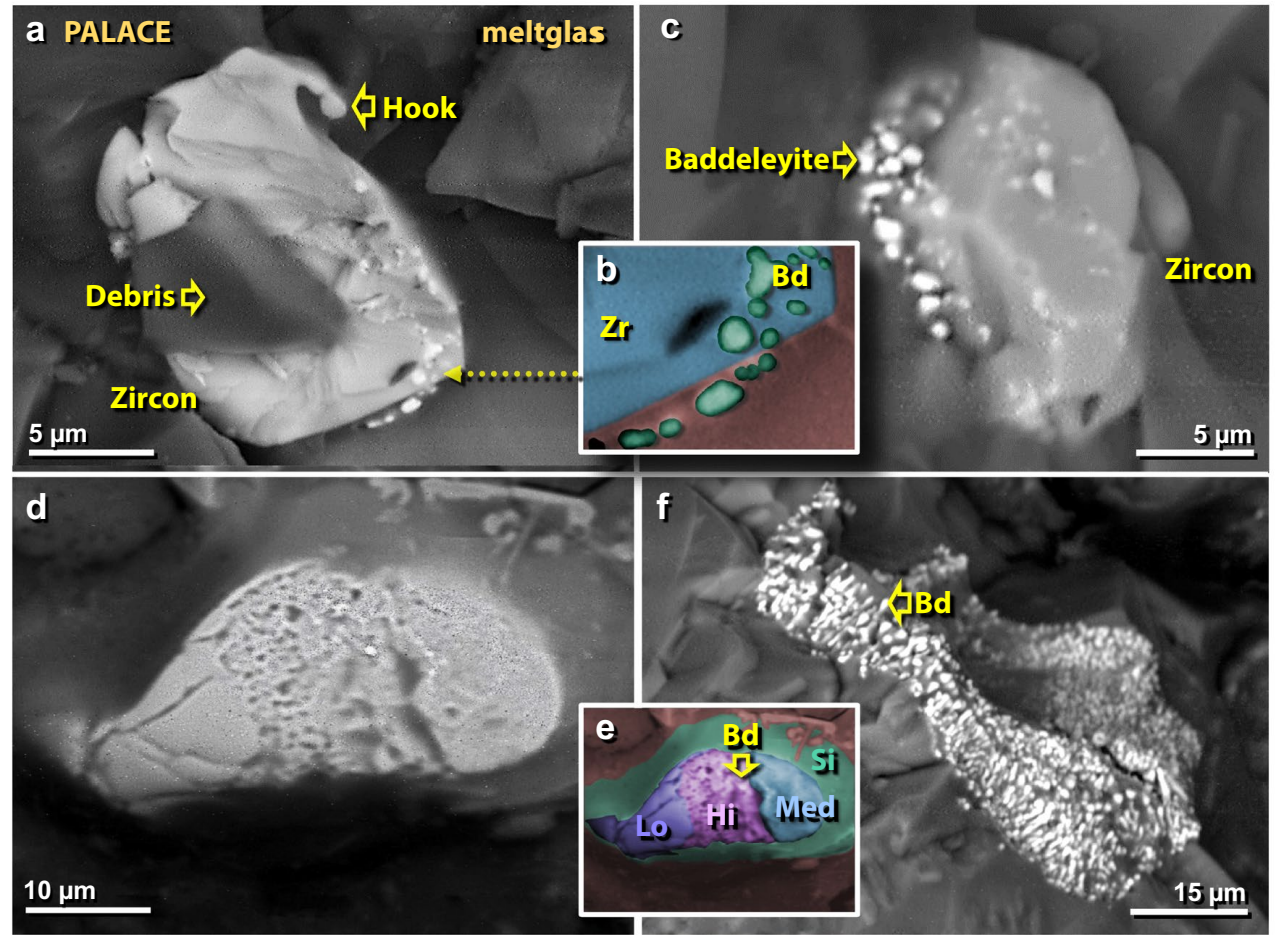
SEM images of melted zircon grains in mudbrick meltglass from the palace. (**a**) Thermally distorted zircon grain with a “hook” that resulted from the flow of molten material at > 1687 °C; the darker area represents unrelated debris on top of zircon. (**b**) Manually constructed EDS-based phase map showing baddeleyite grains (Bd = ZrO_2_) in green, zircon in blue, and melted mudbrick in red. (**c**) Zircon grain showing limited thermal alteration, yet sufficient to cause dissociation into bright baddeleyite grains at ~ 1676 °C. (**d**) Zircon grain exhibiting three phases of thermal alteration, as shown in detail in (**e**), where a manually constructed EDS-based phase map demonstrates that high temperatures caused bubbling in the center band of zircon (purple = Hi) producing sub-micron-sized grains of baddeleyite (e.g., at arrow). Medium temperatures caused zircon to melt and flow (blue = Lo), and lower temperatures at the left end of grain produced thermal cracks (medium blue = Med). The green area marks the high-Si diffusion zone resulting from the dissociation of zircon. (**f**) Zircon grain from TeH has been fully converted to granular baddeleyite.

**Figure 33:**
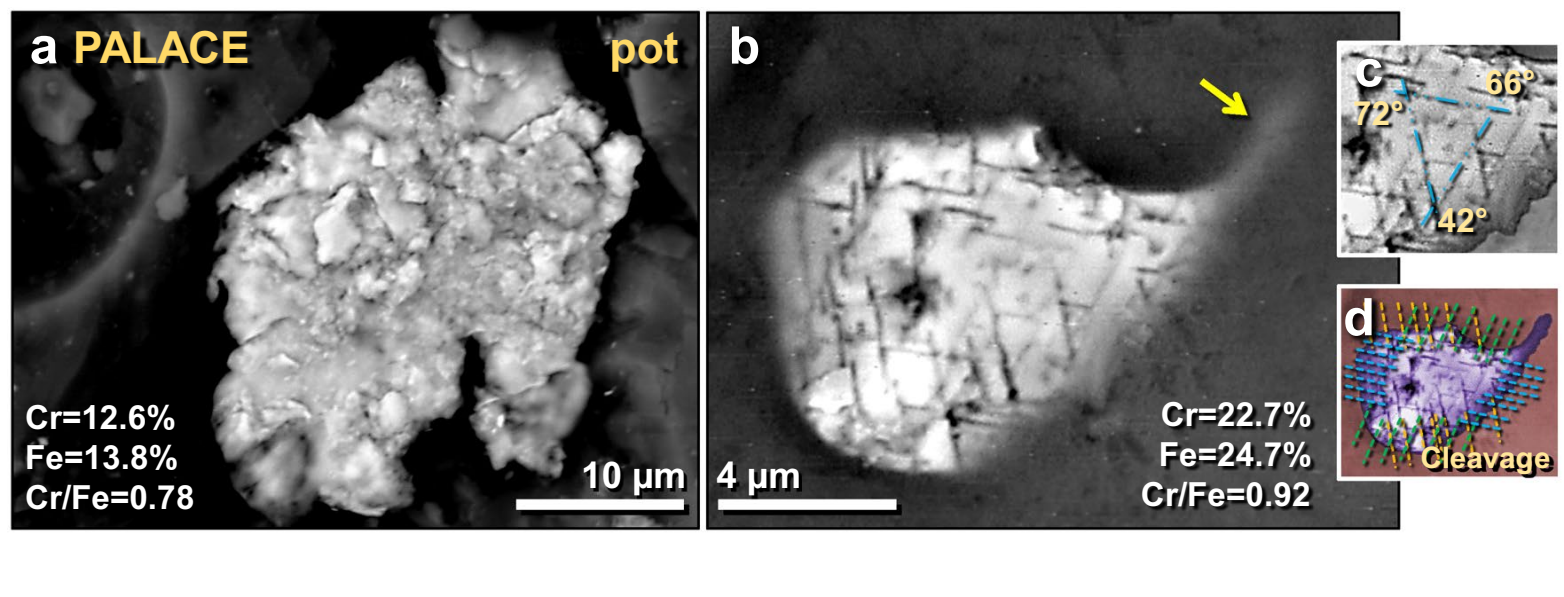
SEM images of melted chromite grains found on a melted potsherd from the palace. (**a**) Shattered, polycrystalline chromite grain that appears to have become agglutinated while molten. (**b**) Melted chromite grain, displaying cleavage (lamellae) suggestive of thermal and/or mechanical shock metamorphism at ~ 12 GPa; (**c**) close-up image showing angles between three sets of crystalline cleavage; (**d**) manually constructed EDS-based phase map showing chromite (purple) embedded in Ca–Al–Si matrix. The lines mark three sets of cleavage extending across the entire grain. A melt tail merging with the matrix is observed to trail off to the upper right of the grain at arrow.

**Figure 34:**
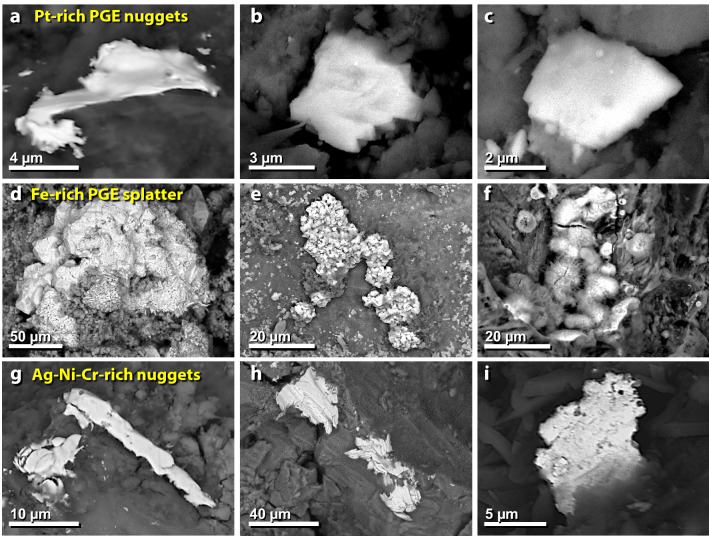
SEM images of nuggets of melted metals in mudbrick meltglass from the palace. (**a**)–(**c**) Pt-dominant TeH nuggets enriched in ruthenium (Ru), rhodium (Rh), palladium (Pd), osmium (Os), iridium (Ir), and platinum (Pt). (**d**)–(**f**) Fe-dominant TeH splatter is also enriched in PGEs. (**g**)–(**i**) Nuggets enriched in varying percentages and combinations of nickel (Ni), chromium (Cr), copper (Cu), and silver (Ag).

**Figure 38:**
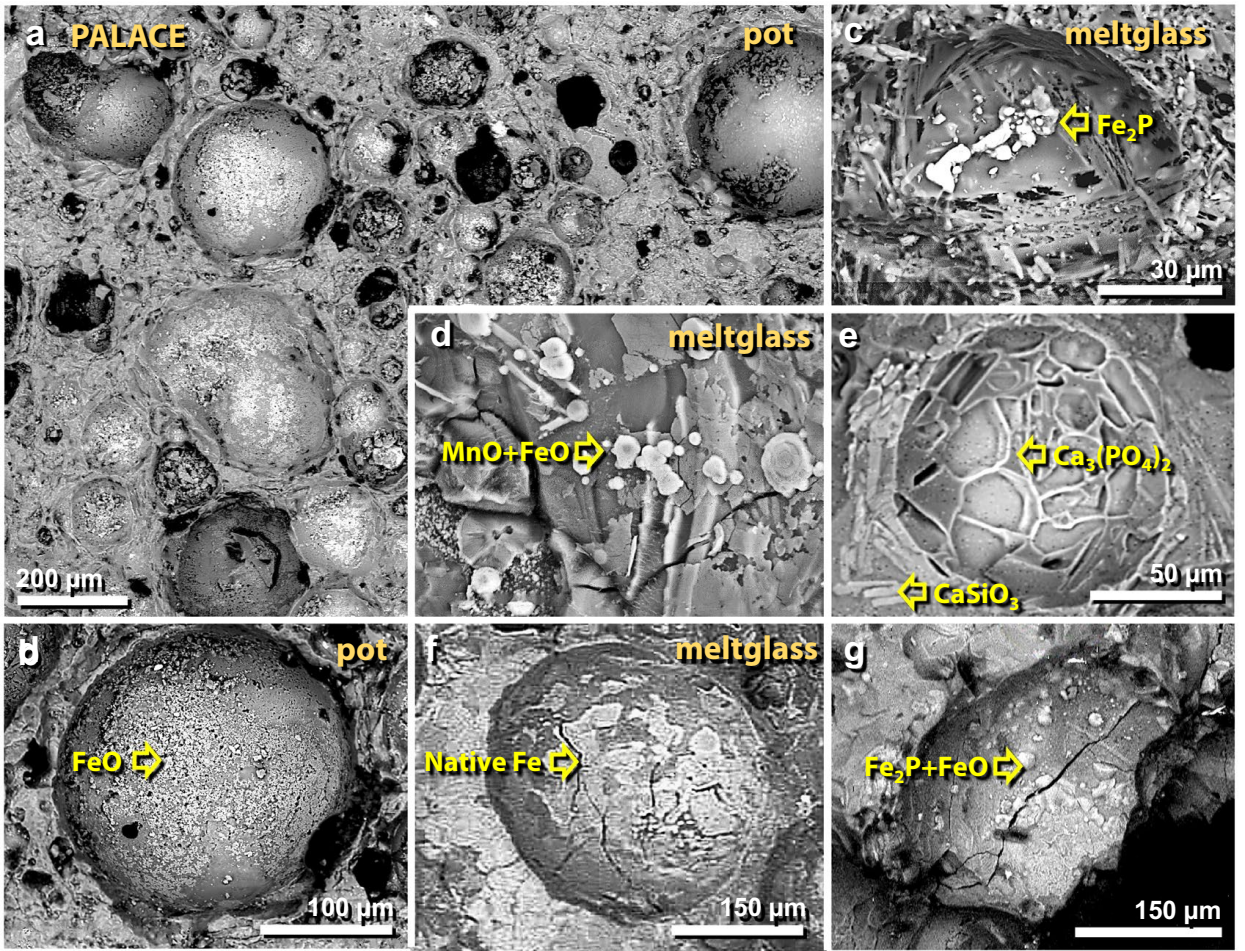
SEM images of gas vesicles in melted material from the palace. (**a**), (**b**) Vesicles in melted pottery are lined with crystals of iron and iron oxide (elemental Fe, Fe_2_O_3_, and/or Fe_3_O_4_). (**c**)–(**g**) Vesicles in melted mudbrick and roofing clay often are lined with a variety of crystals including elemental Fe, iron oxide, Fe phosphide (Fe_2_P), manganese oxide (MnO), calcium phosphate (Ca_3_(PO_4_)_2_), and calcium silicate (CaSiO_3_). These crystals are consistent with vapor deposition at high temperatures.

**Figure 39:**
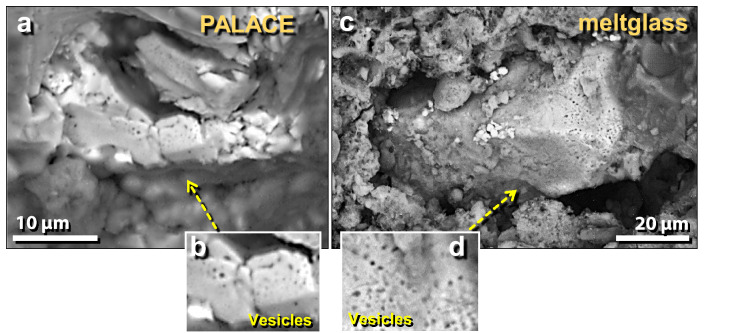
SEM images of melted iron and titanomagnetite in mudbrick meltglass from the palace. (**a**) Shattered elemental Fe grain containing < 0.1 wt.% oxygen as determined by SEM–EDS; (**b**) close-up showing surface porosity. (**c**) Titanomagnetite grain showing surface porosity; (**d**) close-up showing aligned porosity possibly along former grain boundaries. The cause of the porosity is uncertain, but because these grains are associated with other high-temperature melted minerals, we propose that this porosity resulted from exposure to high temperatures.

**Figure 40:**
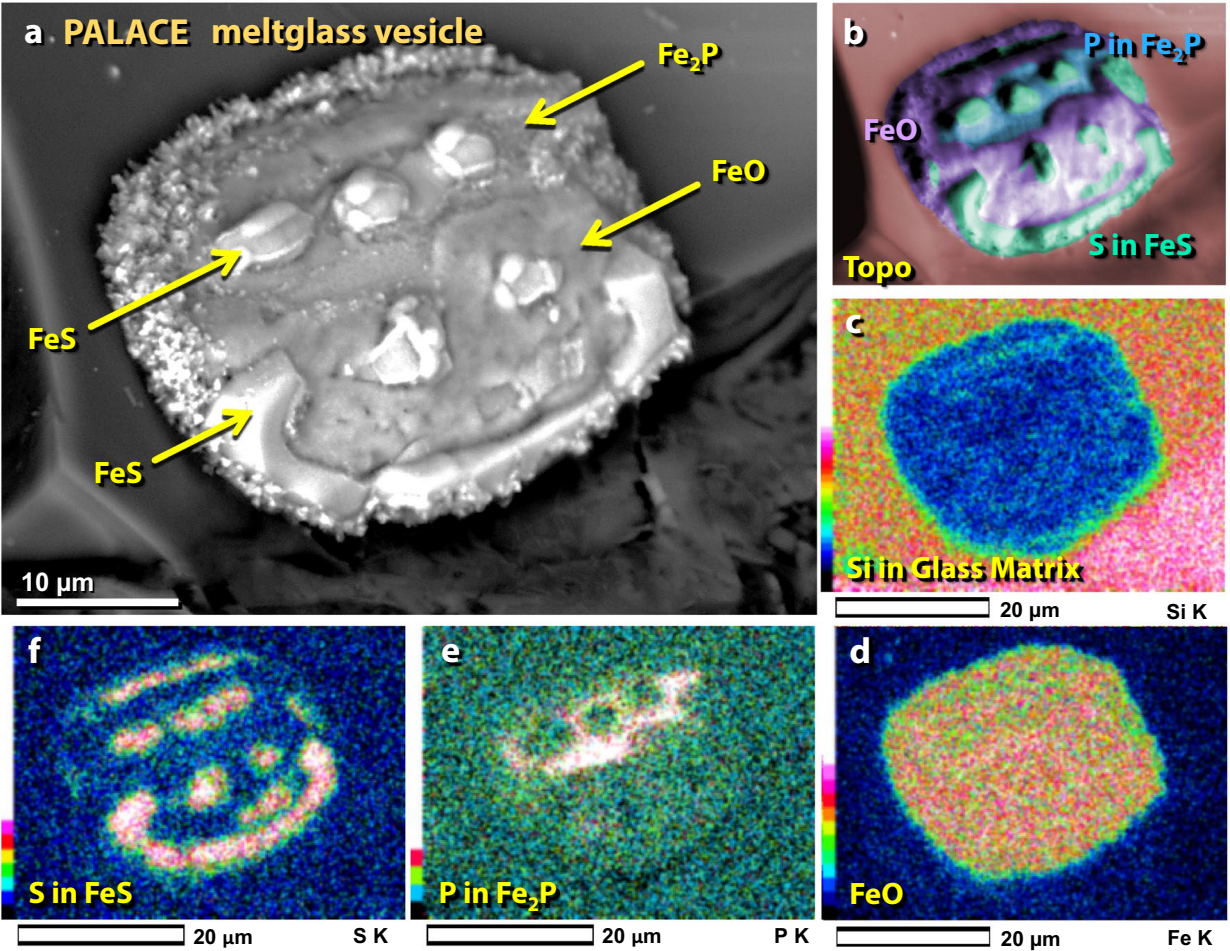
SEM images of Fe-S-P-enriched nugget in vesicle of mudbrick meltglass from the palace. (**a**) Chemically complex nugget inside a vesicle; contains Fe, S, and P. (**b**) Manually constructed EDS-based phase map showing that nugget is dominantly composed of Fe oxides, S as FeS, and P as Fe_2_P. (**c**)–(**f**) SEM–EDS elemental maps showing the composition of nugget by regions.

**Figure 41:**
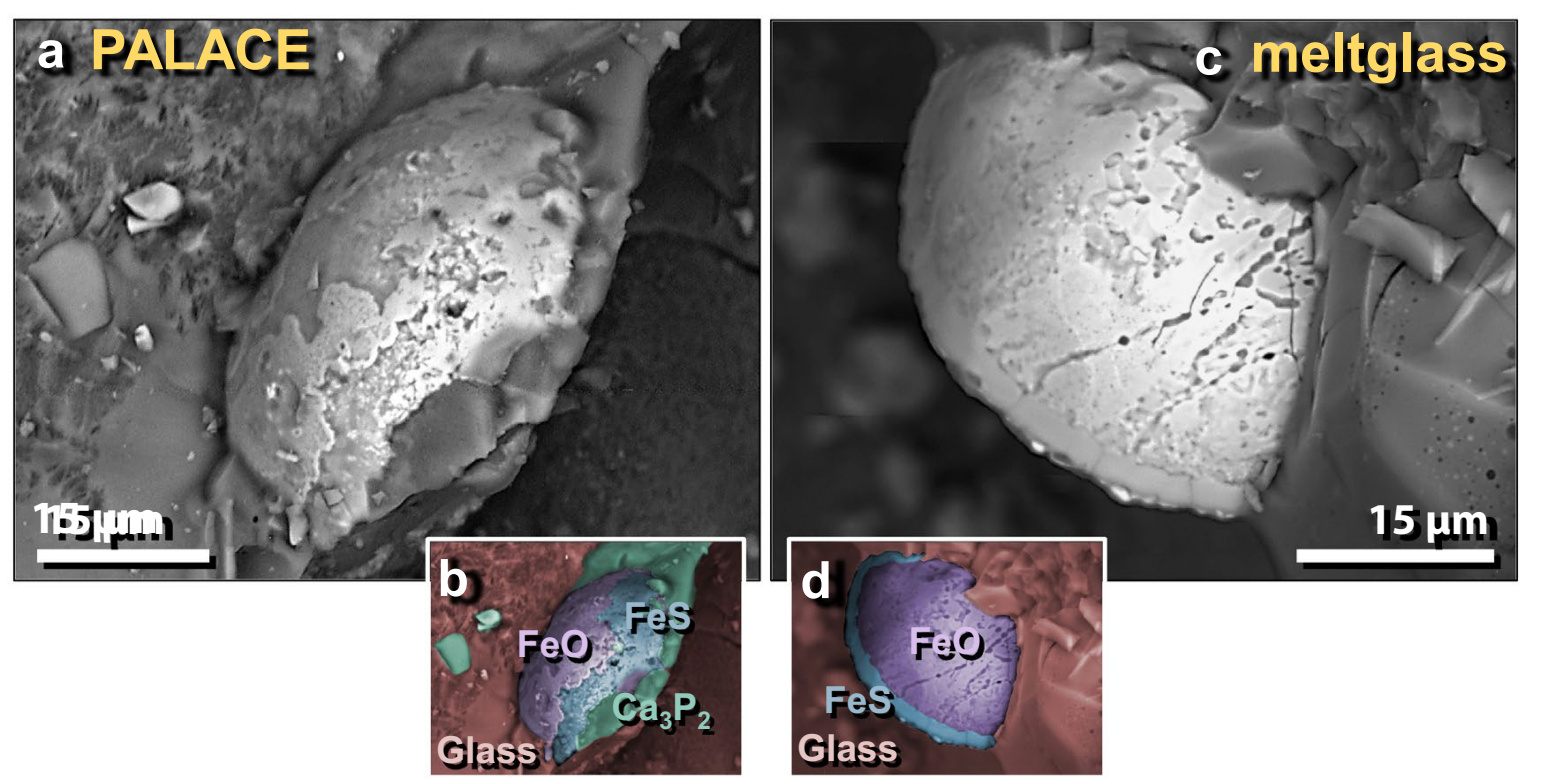
SEM images of Fe–S–Ca–P-rich grains in mudbrick meltglass from the palace. (**a**) Melted Fe-rich grain is chemically complex, containing Fe, S, Ca, and P. (**b**) Manually constructed EDS-based phase map marking areas of Fe oxides (purple, labeled as FeO), FeS (blue), and Ca_3_P_2_, calcium phosphide (green). (**c**) High-temperature melted Fe-rich grain; (**d**) manually constructed EDS-based phase map showing the area that is predominantly Fe oxide, bordered by a thin rim of FeS.

**Figure 42:**
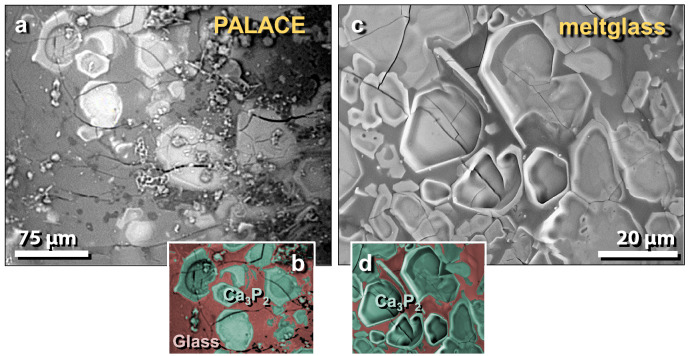
SEM images of calcium phosphide crystals in vesicles of mudbrick meltglass from the palace. (**a**) and (**c**) Crystals of calcium phosphide, Ca_3_P_2_, lining the inside wall of meltglass vesicles; (**b**) and (**d**) manually constructed EDS-based phase map of Ca_3_P_2_ (green) crystals embedded in typical Ca–Al–Si melted matrix.

**Figure 43:**
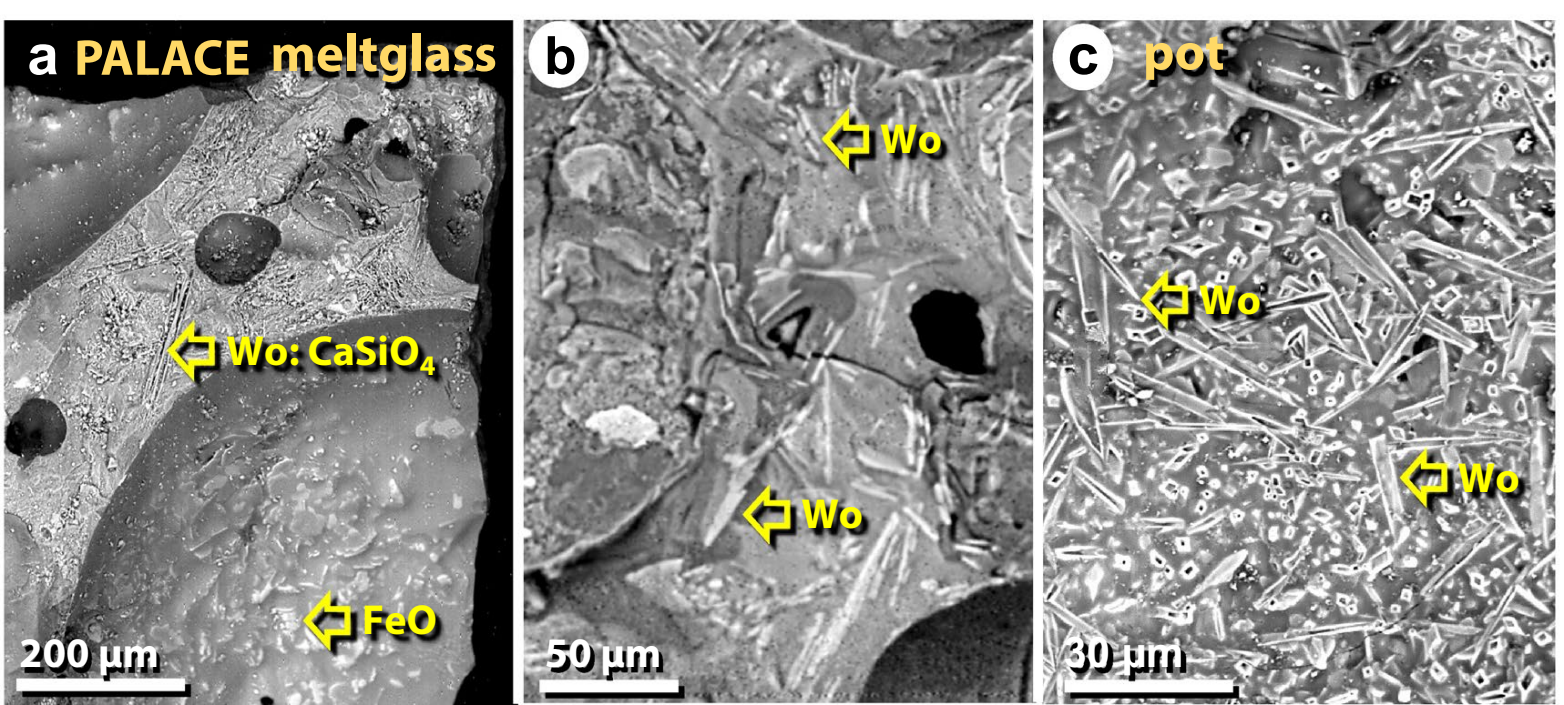
SEM images of wollastonite crystals in palace melted pottery and mudbricks. (**a**) Spindle-like crystals of wollastonite (CaSiO_4_) on the broken face of mudbrick meltglass. Crystals of iron oxide line the vesicle. (**b**) Wollastonite crystal within the meltglass matrix. (**c**) Wollastonite crystals within a vesicle of melted pottery.

**Figure 44:**
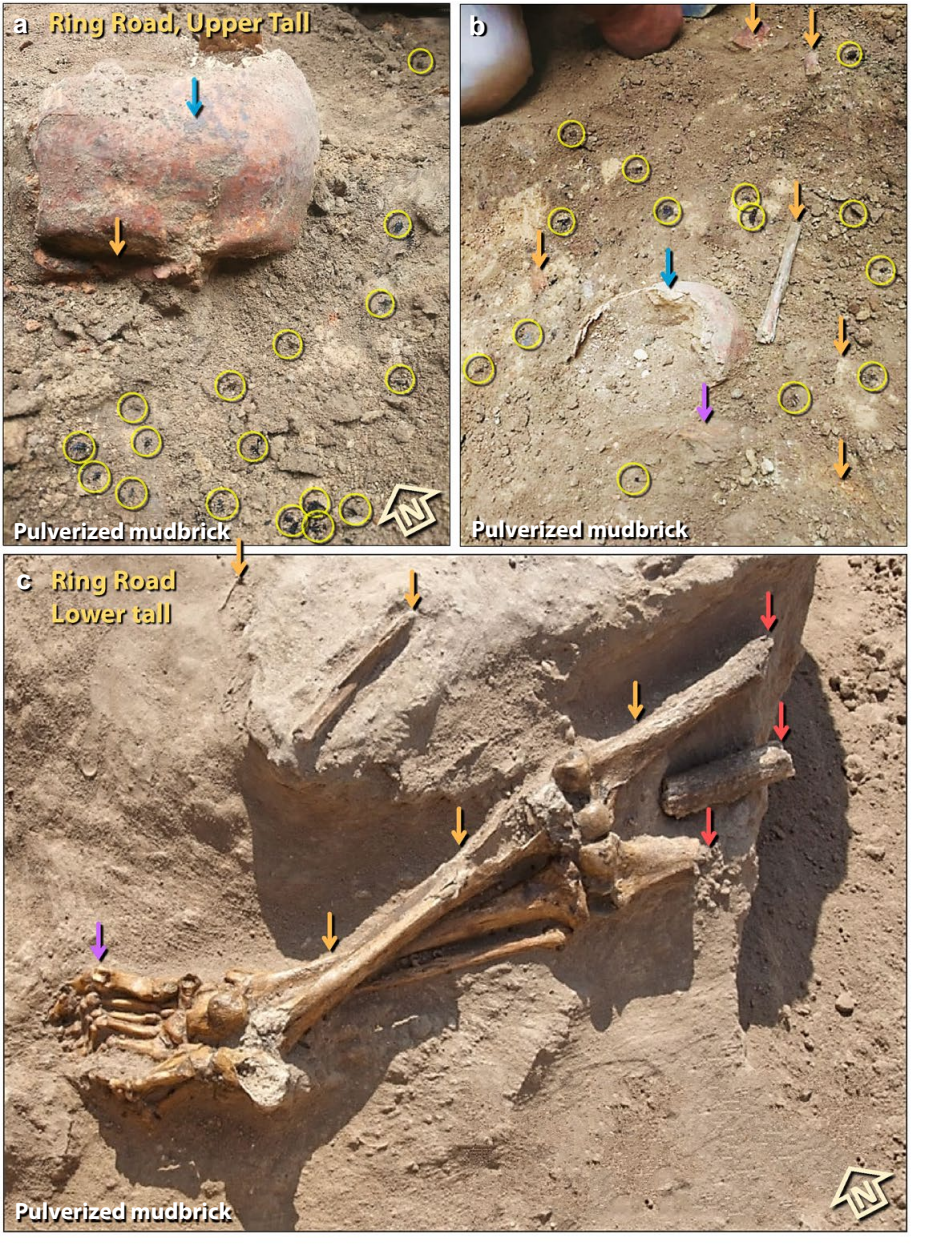
Human bones in the destruction layer. (**a**) Photo of a disarticulated skull found near the palace on the ring road around the upper tall. The right eye socket has been crushed (orange arrow). Skull is embedded in pulverized mudbrick containing numerous charcoal fragments (yellow circles) and is stained with ash commonly found in the destruction layer (blue arrow). The orange tint of the skull suggests it was exposed to temperatures > 200 °C^141^. (**b**) Rear view of the same skull in panel ‘**a**’ (blue arrow) near the second skull (purple arrow) and numerous disarticulated, fragmented human bones (orange arrows). Charcoal fragments at yellow circles. (**c**) Lower torso from the ring road of lower tall (orange arrows), and other disarticulated bones. Bones show evidence of being burned (red arrows); the rest of the skeleton is dismembered and disarticulated. Hyper-flexed toes (purple arrow) are consistent with either perimortem or postmortem exposure to high temperatures.

**Figure 45:**
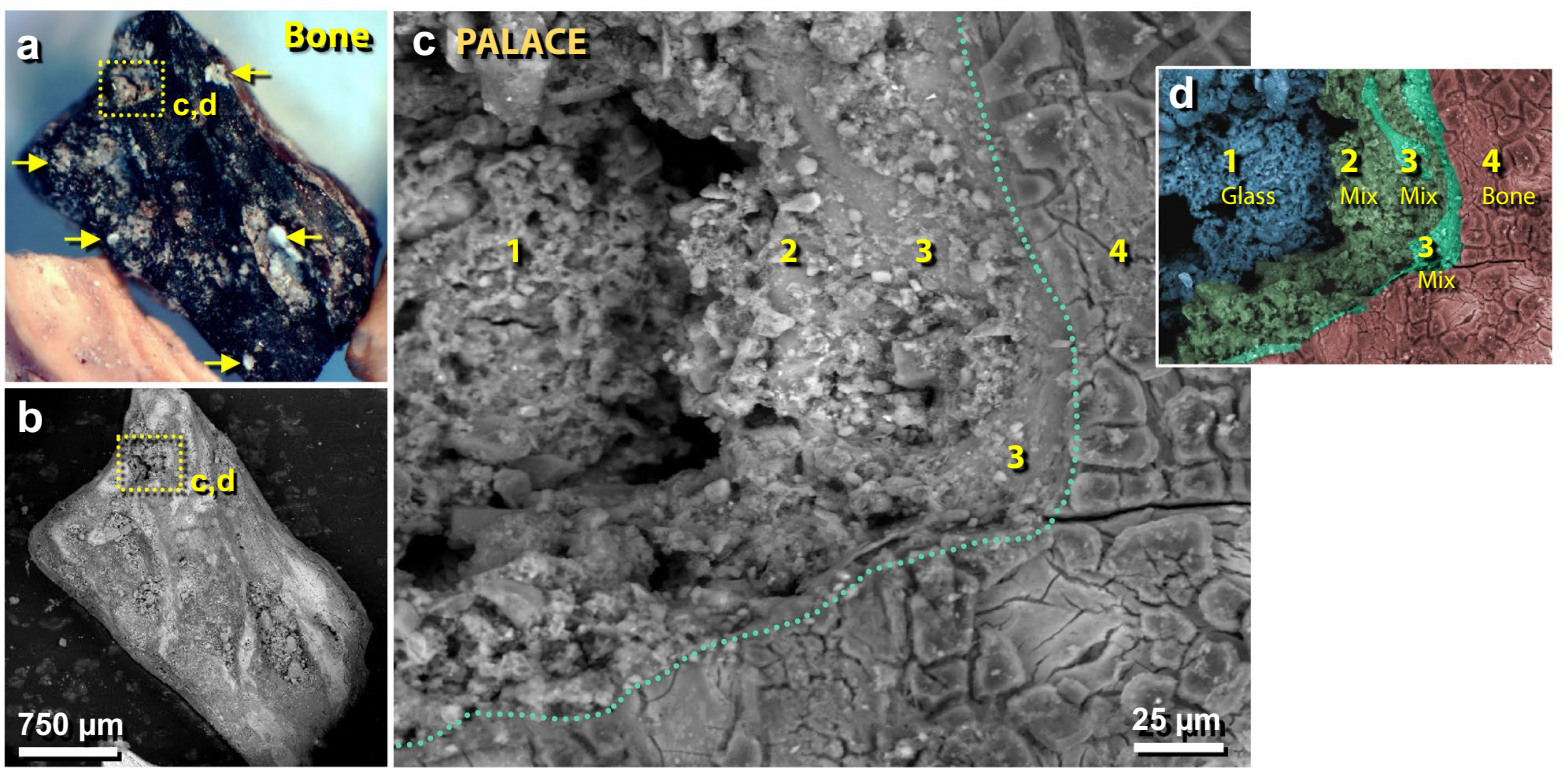
Bone fragment splattered with meltglass in the destruction layer. (**a**) Photomicrograph of 3.5-mm-long charred bone from the palace. The yellow boxed area indicates an area of melted glass on bone, as shown in panels ‘**c**’ and ‘**d**’. Yellow arrows point to other areas with greenish meltglass fused to the bone. (**b**) SEM image of bone in panel ‘**a**’. (**c**) SEM close-up image of boxed area in panel ‘**a**’. Green dotted line marks the glass-to-bone boundary, as further shown in panel ‘**d**’. #1 represents unmelted Ca–Al–Si-rich sediment with no bone component; #2–3 represent partially melted sediment mixed with melted bone (hydroxyapatite); #4 presents charred bone. (**d**) Colorized SEM image of panel ‘**c**’ showing gradation of bone-and-sediment mixing, based on multiple SEM–EDS analyses.

**Figure 46:**
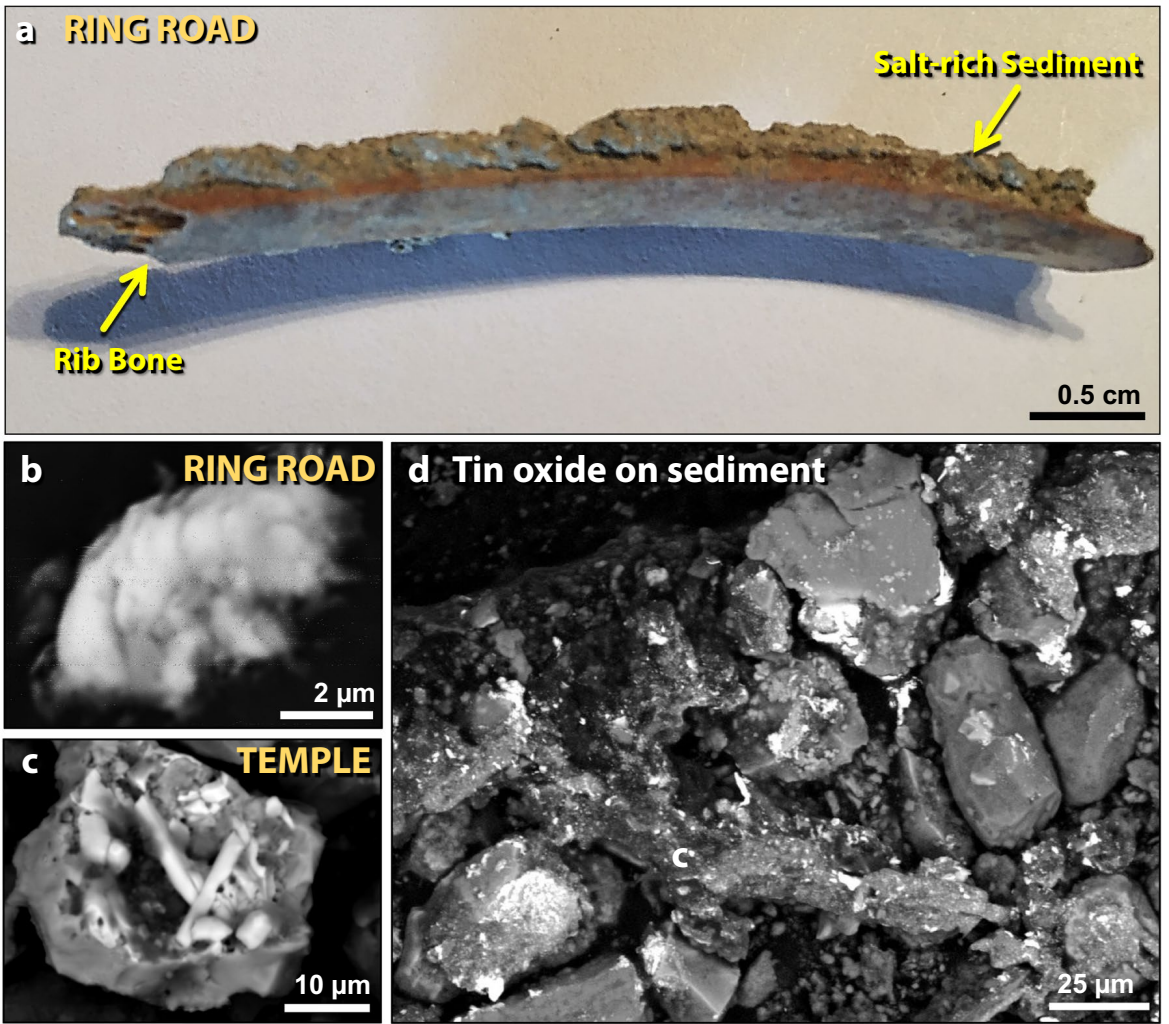
Bone associated with salt and melted tin oxide (SnO_2_). (**a**) Photomicrograph of 4.7-cm-long human or mammal rib bone from the ring road on the lower tall. NaCl is present at high concentrations in the sediment (~ 54 wt.%) and on the bone (~ 46 wt.%). (**b**) Tin oxide particle (SnO_2_) appears to have collided with sediment on the bone while it was molten, possibly as unoxidized tin. The melting point of SnO_2_ is ~ 1630 °C, but unoxidized tin melts at ~ 232 °C. (**c**) A similar tin oxide particle from the temple, ~ 150 m away from the lower ring road sampling site. Particles are fused into meltglass. (**d**) Tin oxide splashed onto sediment around the bone in panel ‘**a**’.

**Figure 47:**
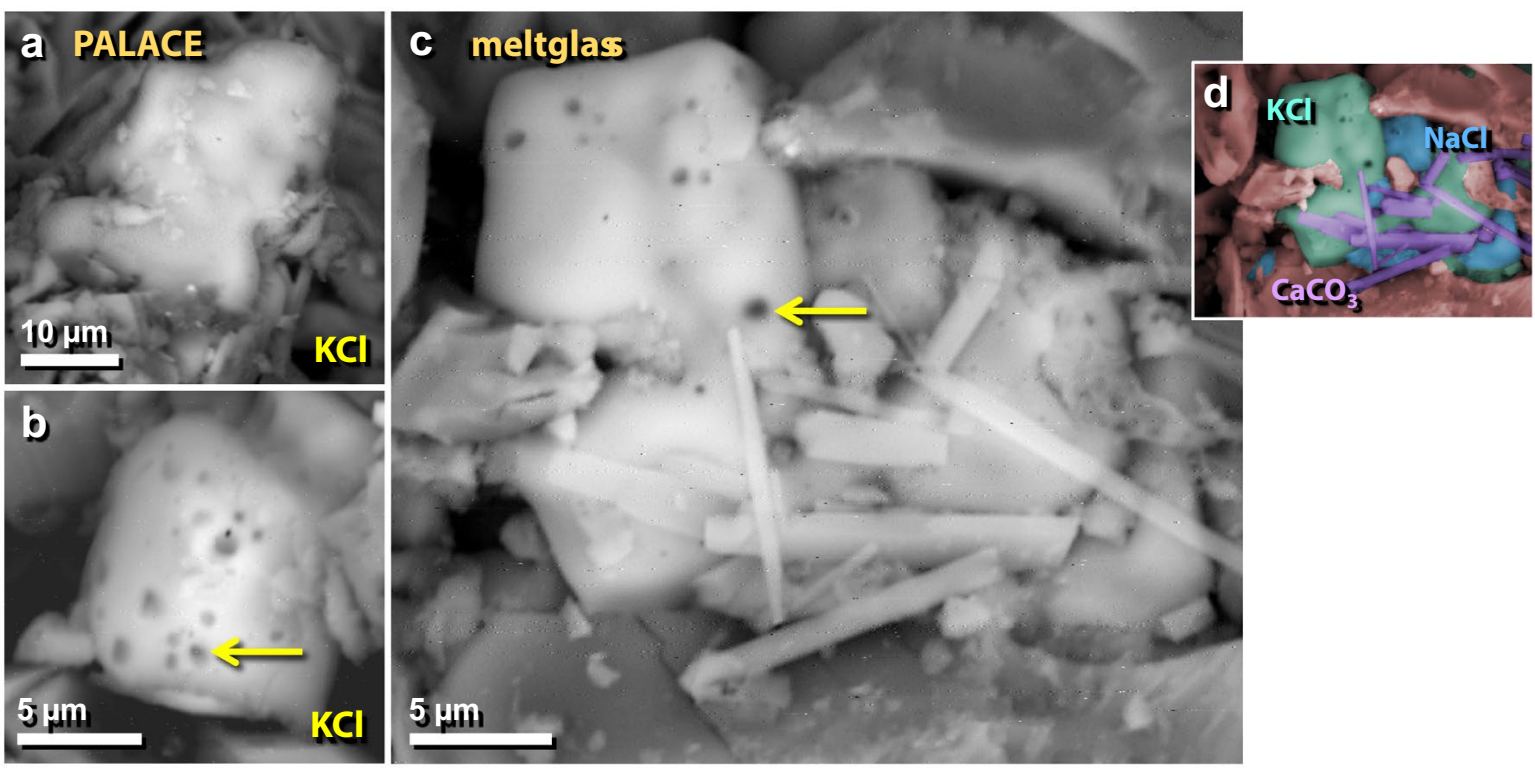
SEM images of melted potassium and sodium salt grains. (**a)**, (**b**) Potassium chloride (KCl) grains melted into the surface of the palace mudbrick meltglass. (**c**) Melted NaCl and KCl grains. Bubbles at arrows suggest the salt grains exceeded their melting points. (**d**) Manually constructed EDS-based phase map of panel ‘**c**’, showing potassium chloride (KCl; green), sodium chloride (NaCl; blue), and spindle-like calcium carbonate crystals (CaCO_3_; purple).

**Figure 51:**
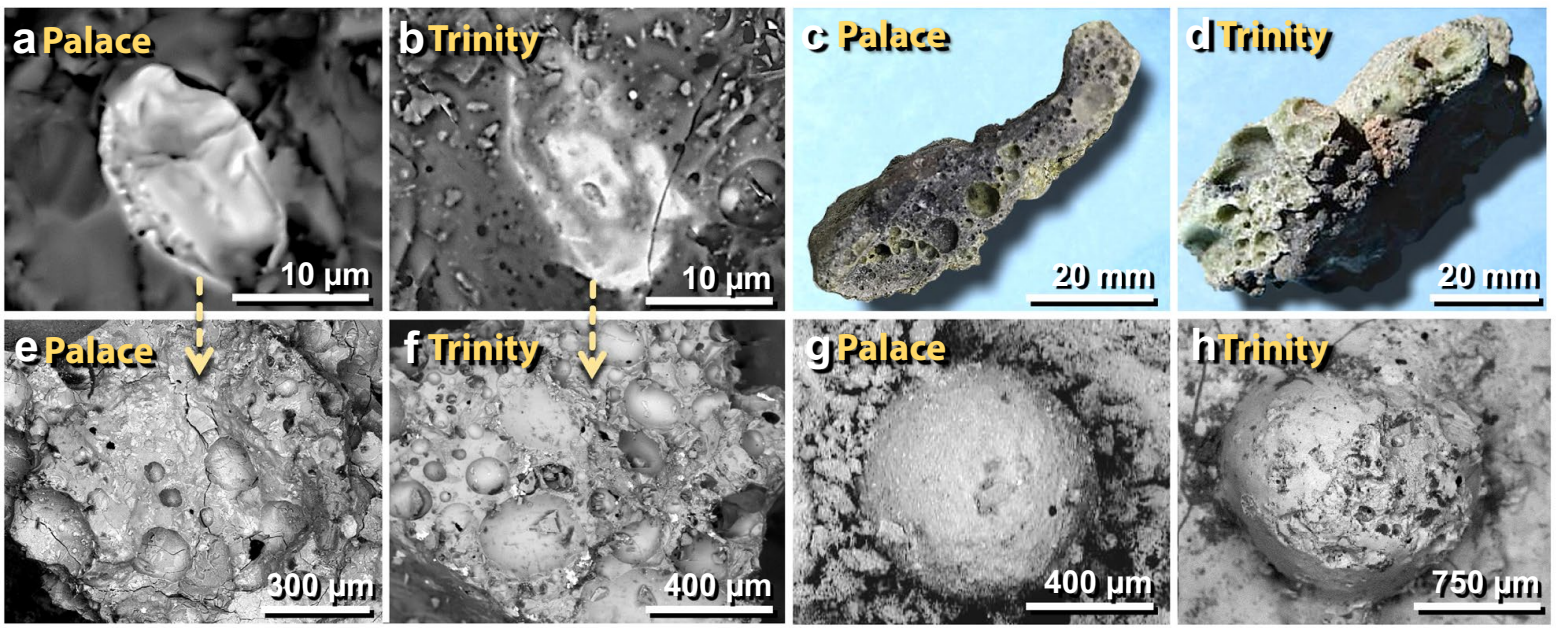
Comparison of melted materials from TeH with those from the Trinity atomic bomb test. (**a**), (**b**) SEM images compare a melted, decorated zircon embedded in mudbrick meltglass from the palace with similar material from Trinity. (**c**), (**d**) Photomicrographs compare a melted potsherd from the palace with similar-looking material from Trinity. (**e**), (**f**) The melted zircons in panels ‘**a**’ and ‘**b**’ were found in highly vesicular glass like these from the palace and the Trinity site, respectively. (**g**), (**h**) SEM images of a spherule fused onto mudbrick meltglass from the palace and a spherule embedded into trinitite from the Trinity atomic test.

Additionally, a further clarification of how the OxCal tool was used are now included in the paper. A new subsection called ‘Bayesian analyses of radiocarbon dates’ was added at the end of the Methods section. This subsection includes two new references, References 203 and 204, which are now added to the Reference list in the published Article:

“There are two functions in OxCal that are used for combining dates from a single inferred event. The ‘R_Combine’ function is used to combine two or more radiocarbon dates from the same source, e.g., a single skeleton^203,204^. The ‘Combine’ function is used to combine two or more radiocarbon dates from different sources that are believed to be coeval, e.g., the date that beams were used to build a cathedral^203,204^. The ‘Combine’ routine is the one used in Bunch et al., because there were different radiocarbon sources, e.g., charred palace beams and charred seeds. The presence of tens of thousands of pieces of charcoal, wood, melted mudbricks, melted pottery, and melted spherules randomly mixed throughout a single unstratified, unconsolidated stratum strongly supports the hypothesis that they represent a single city-wide episode of biomass burning. Thus, the ‘Combine’ function is the appropriate OxCal routine to use.”

The Competing Interests section was updated and now reads:

“T.E.B., M.A.L., J.H.W., W.S.W., G.H., C.R.M., J.P.K., and A.W. volunteer their time as cofounders and/or directors of the Comet Research Group (CRG), a 501(c)(3) nonprofit organization. CRG received donations from the public and contributed funding for equipment, supplies, and scientific analyses. T.E.B., M.A.L., J.H.W., W.S.W., G.H., C.R.M., J.P.K., and A.W. receive no salaries, compensation, stock, or any other financial benefits from CRG, except that M.A.L., G.H., and A.W. realize tax benefits from donations to CRG. In some cases, co-authors have been compensated for out-of-pocket expenses, such as airfare, that are directly related to the TeH research.

P.J.S. volunteers his time and receives no salary from Trinity Southwest University (TSU), a 501(c)(3) nonprofit organization. TSU received donations and contributed support, supplies, equipment, and funding for TeH Excavation Project. P.J.S. was reimbursed by Trinity Southwest University for his hotel room while in Jordan but not for other out-of-pocket expenses.

P.J.S. is the author of two books related to TeH.

A.W. is the author of a book unrelated to TeH.

A.V.A., C.M., D.B., E.C.S., G.K., J.K., K.L., M.C.L.P., R.E.H., S.M., T.D.B., and T.W. received salaries, supplies, equipment, and/or funding for scientific analyses from their respective universities/organizations, which, due to the worldwide publicity, possibly stand to benefit from increased donations and student enrollments.

All co-authors have not yet but may receive reimbursements for attending symposia on this research from their respective organizations. All co-authors were involved in various aspects of conceptualization, design, data collection, analysis, the decision to publish, and/or preparation of the manuscript.”

Finally, the beginning of the Acknowledgements section was updated,

“We gratefully acknowledge the invaluable assistance of the senior staff at Tall el-Hammam: Director, Steven Collins; Assistant Director, Gary A. Byers, and Assistant Director, Carrol M. Kobs.”

now reads:

“We gratefully acknowledge the invaluable assistance of the senior staff at Tall el-Hammam: Director, Steven Collins; Assistant Director, Gary A. Byers, and Assistant Director, Carrol M. Kobs. Additionally, we thank Daniel Galassini, Michael Luddeni, Brandy Forrest, James Barber, and Sultan Madi, who took the site excavation photographs for the Tall el-Hammam Excavation Project, which made them available for this study (Fig 1B, Figs 4D & 4E, Fig 6, Figs 7A & 7B, Fig 15A, and Fig 44C—Michael Luddeni, Fig 3—Daniel Galassini, Figs 7C & 7D, Fig 15B—Brandy Forrest, Fig 16A—James Barber, Figs 44A & 44B—Sultan Madi).”

The original Article has been corrected.
